# Structural Insights into *Saccharomyces cerevisiae* Msh4–Msh5 Complex Function Using Homology Modeling

**DOI:** 10.1371/journal.pone.0078753

**Published:** 2013-11-14

**Authors:** Ramaswamy Rakshambikai, Narayanaswamy Srinivasan, Koodali Thazath Nishant

**Affiliations:** 1 Molecular Biophysics Unit, Indian Institute of Science, Bangalore, India; 2 School of Biology, Indian Institute of Science Education and Research, Thiruvananthapuram, India; Wake Forest University, United States of America

## Abstract

The Msh4–Msh5 protein complex in eukaryotes is involved in stabilizing Holliday junctions and its progenitors to facilitate crossing over during Meiosis I. These functions of the Msh4–Msh5 complex are essential for proper chromosomal segregation during the first meiotic division. The Msh4/5 proteins are homologous to the bacterial mismatch repair protein MutS and other MutS homologs (Msh2, Msh3, Msh6). *Saccharomyces cerevisiae msh4/5* point mutants were identified recently that show two fold reduction in crossing over, compared to wild-type without affecting chromosome segregation. Three distinct classes of *msh4/5* point mutations could be sorted based on their meiotic phenotypes. These include *msh4/5* mutations that have a) crossover and viability defects similar to *msh4/5* null mutants; b) intermediate defects in crossing over and viability and c) defects only in crossing over. The absence of a crystal structure for the Msh4–Msh5 complex has hindered an understanding of the structural aspects of Msh4–Msh5 function as well as molecular explanation for the meiotic defects observed in *msh4/5* mutations. To address this problem, we generated a structural model of the *S. cerevisiae* Msh4–Msh5 complex using homology modeling. Further, structural analysis tailored with evolutionary information is used to predict sites with potentially critical roles in Msh4–Msh5 complex formation, DNA binding and to explain asymmetry within the Msh4–Msh5 complex. We also provide a structural rationale for the meiotic defects observed in the *msh4/5* point mutations. The mutations are likely to affect stability of the Msh4/5 proteins and/or interactions with DNA. The Msh4–Msh5 model will facilitate the design and interpretation of new mutational data as well as structural studies of this important complex involved in meiotic chromosome segregation.

## Introduction

The MutS homodimer in bacteria is involved in the repair of mismatches that occur during DNA replication [1]. The MutS homologs in eukaryotes form heterodimeric complexes with each other except Msh1. MutSα (Msh2–Msh6) heterodimeric complex is required for repair of mismatches and small (1–2 base) insertion/deletion (in/del) loops that arise during DNA replication [2]. The MutSβ (Msh2–Msh3) complex repairs some single base in/del loops and loops that are two bases or larger [2]. MutSγ (Msh4–Msh5) does not participate in repair of mismatches or in/del loops [3]. Instead this complex plays a critical role in ensuring meiotic crossover formation and segregation of homologous chromosome pairs [3], [4], [5], [6].

**Figure 1:**
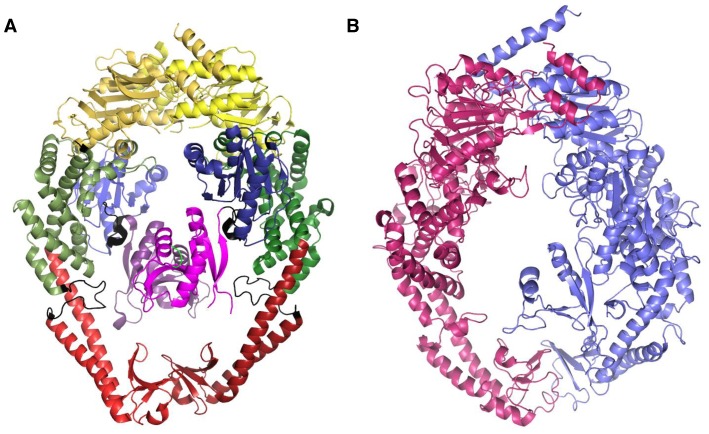
Structure of *E. coli* MutS homodimer and a model of the *S. cerevisiae* Msh4–Msh5 complex. A) *E. coli* MutS homodimer showing five domains. Domain I is colored in magenta, domain II is colored in blue, domain III in green, domain IV in red and domain V in yellow. The connecting regions are colored in black. B) Cartoon representation of the modeled complex of *S. cerevisiae* Msh4 and Msh5. Msh4 is colored in purple and Msh5 in magenta.

The MutS homodimer has the shape of an oval disk with two channels of dimensions ∼30×20 and ∼40×20 Å with DNA passing through the larger channel [1], [7]. Each subunit of the MutS protein comprises of five structural domains (Figure 1A). Domains I and IV bind mismatch DNA and the domain V contains ATP/ADP nucleotide binding sites. Domain I is also involved in mismatch recognition using the conserved Phe-X-Glu motif [2]. The DNA and nucleotide binding domains are connected by domain III. Domain III connects with domain IV directly and connects with domain I through the uncharacterized domain II. These domains are also conserved in the MutSα and MutSβ homologs. MutSγ has homology with domains II, III, IV and V but lacks the N terminal domain I. Absence of domain I is expected to result in a large single channel of dimensions 70×30 Å and inability to bind mismatch DNA during replication [1], [3]. Instead the Msh4/5 proteins serve as pro-crossover factors during meiotic recombination. Physical, biochemical, genetic and cytological studies have illuminated several aspects of Msh4–Msh5 function in meiotic crossing over as outlined below.

In *Saccharomyces cerevisiae* meiotic crossovers are initiated by the programmed introduction of ∼140–170 DNA double strand breaks (DSBs) by the Spo11 protein in combination with accessory factors [8], [9], [10]. Physical assays performed in *S. cerevisiae* have provided molecular details into the sequence of events during repair of DSBs into crossover products [11], [12], [13], [14], [15]. DSBs are processed by endo and exonucleases to produce 3′ single stranded DNA [16], [17], [18]. Dmc1 and Rad51 proteins form nucleoprotein filaments on the 3′ single stranded DNA and catalyse strand invasion into homologous duplex DNA [19], [20]. The nascent strand invasion matures into a single end invasion intermediate (SEI). For DSBs that are repaired as interfering crossovers, the SEI intermediate is thought to be stabilized by the Msh4–Msh5 complex and form double Holliday junctions (dHJ) by capture of the second DSB end. Resolution of dHJ into crossovers is facilitated by Msh4–Msh5 in association with other repair factors [21], [22], [23]. These functions of Msh4–Msh5 complex are summarized in a simple model by Snowden et al., [21].

Consistent with the physical studies, genetic and biochemical data support the role of the Msh4–Msh5 complex in meiotic crossover formation. *S. cerevisiae msh4Δ*, *msh5Δ* mutants have strong defects in meiotic crossing over (2.5 fold decrease), spore viability (30–40%) and disjunction of homologous chromosomes [3], [4], [24]. Mutations of these genes in male and female mice cause chromosome pairing and synapsis defects and result in sterility [25], [26], [27]. In humans, non-disjunction of homologous chromosomes during meiosis is associated with infertility, and congenital birth defects (such as Down syndrome) [28]. Biochemical studies have shown that the hMSH4–hMSH5 complex specifically binds to Holliday junction DNA and its progenitors that are key intermediates during crossover formation [21]. hMSH4–hMSH5 is thought to form multiple sliding clamps on these substrates and stabilize them. Biochemical data also suggest that the hMSH4 protein interacts with the MutL homologs hMLH1 and hMLH3 [29], [30]. These data are supported by cell biological observations in mammals that suggest a subset of the Msh4–Msh5 complexes stabilizing Holliday junctions interact with the Mlh1, Mlh3 proteins [29], [30], [31], [32], [33]. The Mlh1 foci on pachytene chromosomes are known to correspond to future crossover sites [34], [35], [36].

The Msh4–Msh5, Mlh1–Mlh3 complexes are part of the major crossover pathway in *S. cerevisiae* and mammals. A smaller subset of crossovers in these organisms is made through the Mus81-Mms4 pathway [24], [37], [38], [39]. The central role played by the Msh4–Msh5 complex in meiotic crossing over encouraged a detailed mutational study of these proteins for meiotic crossover and chromosome segregation defects [6]. It was observed that the Msh5 protein is more sensitive to mutations and *msh5* mutants showed more severe phenotypes compared to *msh4* mutants. *msh4/5* mutations were classified into three types based on crossover frequency and spore viability. These include a) mutations with spore viability and crossover frequency similar to that of *msh4/5* “null” mutations, b) mutations with intermediate defects in spore viability and crossing over and c) mutations with only crossover defects. Interestingly, mutations in equivalent positions in Msh4 and Msh5 ATPase and DNA binding domains were observed that had asymmetric effects on crossover frequency and spore viability.

The aim of this study is to provide a structural basis for understanding Msh4–Msh5 function as well as molecular explanation for each of these *msh4/5* mutations. As no crystal structure is available for the Msh4–Msh5 complex, homology modeling was used to generate a structural model for this complex using the hMSH2–hMSH6 crystal structure as the template. Homology modeling has proved to be useful in a number of cases where crystal structures are not available for a protein [40], [41]. The modeling studies suggest that the *msh4/5* mutations result in meiotic defects by two mechanisms: by affecting stability of the Msh4/5 proteins or interaction of the Msh4–Msh5 complex with the DNA. The model has not only been used to explain the structural basis of the meiotic defects observed in the mutations but also to propose further mutations that may be analyzed. These include residues at the putative interface of the Msh4–Msh5 complex, residues that may be involved in DNA binding and double mutations that may serve as compensatory mutations. Such information is useful to predict incompatibilities between segregating polymorphisms in *MSH4* and *MSH5* genes in populations. More generally the availability of a model for Msh4–Msh5 structure will facilitate the design of new mutational studies of the complex, interpretation of *MSH4/5* polymorphism data in populations and a mechanistic understanding of Msh4–Msh5 function in meiotic crossing over.

## Results and Discussion

### Homology modeling of the Msh4–Msh5 complex

Crystal structures of bacterial MutS, and eukaryotic MutSα and MutSβ complexes are available [1], [42], [43], [44]. The structural information is useful for providing explanations for the phenotypic effect of mutations in these proteins. It also enables prediction of important residues and domains that may compromise the protein function if mutated. We built a homology model of *S. cerevisiae* Msh4–Msh5 based on alignment with the hMSH2–hMSH6 complex (PDB code 2o8b) as the template. Alignments between the templates and targets obtained from automatic programs are considered unsatisfactory. This is because, automatic programs tend to introduce breaks in the middle of regular secondary structural elements, or align hydrophobic residues in the target with solvent exposed residues in the template among other reasons. Extensive manual intervention was therefore required to arrive at a high quality alignment suitable for the comparative modeling. Reasons for choosing hMSH2–hMSH6 complex as the template are discussed below.

The choice of using hMSH2–hMSH6 as the template was intentional based on the rigorous analysis of the quality of alignment between Msh4 & hMSH2, Msh4 & hMSH6, Msh5 & hMSH2, Msh5 & hMSH6 and Msh4 & hMSH3 pairs. Poor sequence identity of the order of 20% amongst all pairs meant that templates could not be decided purely based on sequence identity. Therefore all possible alignments were assessed to decide the template. Figure S1 provides a structure based alignment between Msh4, hMSH3 and hMSH6. This alignment has been generated by first structurally aligning hMSH3 and hMSH6 and then aligning Msh4 to this alignment. From the alignment, considering insertions and deletions (in/dels) unique to hMSH3 or hMSH6 individually, hMSH3 shows more in/dels than hMSH6 when both proteins are aligned with Msh4. Particularly one of the deletions in hMSH3 which spans to about 25 residues is problematic considering that ab-initio modeling of such long stretches is likely to be rather inaccurate. In addition, a number of observations suggest recognition and repair of mispairs as well as general DNA binding occurs through conserved mechanism in hMSH2–hMSH6 and MutS, but is substantially different in case of hMSH2–hMSH3 [42], [45], [46], [47], [48], [49], [50]. Thus, we decided to use the hMSH2–hMSH6 template for modeling Msh4–Msh5 structure. We modeled Msh4 with hMSH6 as the template and Msh5 with hMSH2 as the template.

The curated alignment obtained has been provided in Figure S2. The decision on the choice of the template was influenced by the quality of alignments which are discussed below.

It was observed that there existed more cases of in/dels within regular secondary structures in case of hMSH2 as template for Msh4 and hMSH6 as template for Msh5. The assessment of the alignment involved the use of structural environments around the sequence. Also, features such as solvent accessibility and secondary structure of residues were considered. For example, buried apolar residues replaced by buried polar residues and exposed polar residues being replaced by exposed apolar residues were commonly seen when Msh4 was aligned with hMSH2 and Msh5 with hMSH6 as the template. So, if the templates are swapped in modeling Msh4 and Msh5, not only was the quality of alignment poor, the modeled structure had large number of short contacts and collapsed during the energy-minimization steps. Two non-bonded atoms are said to be in short contact if their inter-atomic distance is too short in comparison to the classic contact criteria proposed by Ramachandran and coworkers [51]. The modeled structure of Msh4–Msh5 complex is represented as a cartoon in Figure 1B. The residues that could not be modeled due to in/dels in the alignment are indicated in Table 1. Correspondence between amino acid position in the Msh4, Msh5 protein sequences and the modeled structure are shown in Tables S1 and S2.

**Table 1:** In/dels in Msh4 and Msh5 that could not be modeled.

Protein	In/del number	Residues part of in/dels
**Msh4**	1	1–58
	2	172–174
	3	272–275
	4	688–696
	5	846
	6	901
**Msh5**	1	115
	2	204
	3	264
	4	449
	5	510
	6	607–610
	7	678–686
	8	768–774
	9	837–878

### Structural insights from the Msh4–Msh5 model

The Msh4–Msh5 model was used to address asymmetry of the Msh4 and Msh5 subunits within the complex, to map interface residues between Msh4 and Msh5 and to analyze the interaction of the complex with Holliday junction DNA. These are discussed in further detail below.

#### Asymmetry in the Msh4–Msh5 complex

**Figure 2:**
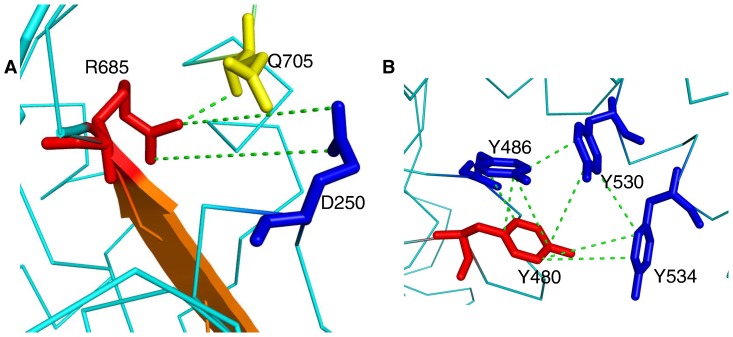
Molecular interactions of Msh5 residues involved in ATP hydrolysis and DNA binding. A) Side chain interactions of R685 with Q705 and D250 stabilize the β-sheet preceding R685. B) Aromatic-aromatic interactions between Y480, Y486, Y530 and Y534. Interactions are shown in dashed green lines.

Subunits of the MutS and MutSα complexes show asymmetry for mismatch binding and ATP hydrolysis [1], [42], [45], [46], [52], [53], [54], [55]. A similar functional asymmetry has also been observed for the Msh4–Msh5 complex in the ATPase and DNA binding domains [6], [56]. For example, both the ATP binding mutant alleles, *msh4 G639A* and *msh5 G648A* have spore viability similar to *msh4/5Δ* mutants (Table S3) [6]. But the ATP hydrolysis domain mutant *msh4 R676W* has wild-type spore viability while the equivalent mutation in *msh5 R685W* shows null phenotype. Similarly mutant alleles in the DNA binding domain, *msh4 N532A*, *Y485A*, *L493A*, and *L553A* have spore viabilities of 89, 95, 75 and 95%, respectively compared to equivalent mutations in *msh5 D527A* (30%), *Y480A* (67%), *V488A* (40%), and *L548A* (50%) respectively. Reasons for their asymmetric phenotypes are outlined below. The ATP binding mutations *msh4 G639A* and *msh5 G648A* are poorly tolerated in both Msh4 and Msh5 because of structural constraint. The Glycine residues have positive φ values which is not comfortably adopted by non-Glycine residues. This is also indicated by the high conservation of these residues. The ATP hydrolysis residue, Msh5 R685 is involved in main chain hydrogen bonding to stabilize the β sheet as shown in Figure 2A and is relatively more crucial than Msh4 R676. The *msh5 R685W* mutation is therefore poorly tolerated compared to the equivalent mutation *msh4 R676W*. In the DNA binding domain, Msh5 D527 is a solvent exposed residue and hence leads to instability when mutated to a hydrophobic residue such as Alanine. The Msh5 Y480 in the DNA binding domain is involved in aromatic interactions as shown in Figure 2B which is not satisfied when mutated to Alanine. There are no such strong constraints for Msh4 N532 and Y485. Mutations of these Msh4 residues are therefore tolerated better. The hMSH4–hMSH5 complex specifically binds to Holliday junction DNA compared to linear ds DNA or other branched DNA structures [21]. Asymmetry in the DNA and ATP binding domains of the Msh4–Msh5 complex might reflect different roles for Msh4 and Msh5 in recognition and binding of Holliday junction DNA.

#### Binding to Holliday junctions

**Figure 3:**
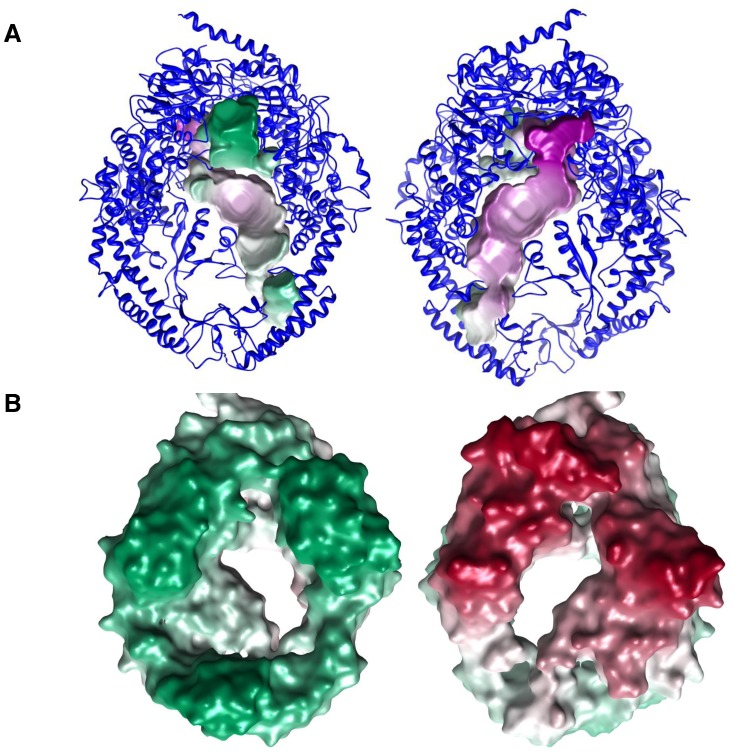
Msh4–Msh5 model highlighting the binding cavity for the Holliday junction. Panel A shows the cartoon representation and Panel B shows the surface representation of the model.

Volume of the cavity in the Msh4–Msh5 modeled structure was calculated to be 16676 Å^3^. The images of the cavity are shown in Figure 3. We also estimated the volume of the Holliday junction in square planar conformation to be 6228 Å^3^. The Holliday junction is known to take up stacked conformations in the presence of metal ions [57], [58]. The volume measurements of the central cavity of the Msh4–Msh5 complex are consistent with the dimensions for a square planar geometry or other conformations of the Holliday junction. However it is not possible to decisively conclude the exact conformation or nature of binding of the Holliday junction to the Msh4–Msh5 complex on the basis of these studies. The probability of various Msh4/5 residues to bind to the DNA based on prediction by the Multi-VORFFIP (MV) server is indicated in Table S4 [59]. A probability of greater than 0.7 indicates a higher chance of being able to interact with the DNA. Out of thirty seven residues in Msh4 and ten residues in Msh5 showing a probability greater than 0.7, eight residues in Msh4 were mapped to be in the DNA binding domain. None of the residues having probability of greater than 0.7 in Msh5 map to the DNA binding domain. These results also suggest differences between Msh4 and Msh5 in DNA binding. DNA binding residues were also predicted on the basis of a structure based sequence alignment using Msh proteins from human and yeast. The DNA binding residues known in the literature and that predicted by the Multi-VORFFIP (MV) server were compared [42], [44], [59]. The two categories of residues are marked in Figure S3. Among the residues mentioned in literature, Msh4 H73 has a relatively high probability of 0.66 while other residues show a lower probability. In addition, two residues, Msh5 L155 and Msh5 I254 are conserved across the MSH family further confirming their role in DNA binding. From the residues predicted by the MV server, most residues are not well conserved and therefore seem to be specific to the Msh4–Msh5 complex. However, two residues, Msh5 D104 and Msh5 N347 are conserved in the MSH family and hence are likely to be important for DNA binding.

#### Msh4–Msh5 interface residues

Most probable Msh4–Msh5 interface residues were identified as discussed in Materials and Methods. They include residues within the ATPase domain in Msh4 (745–820 aa) and Msh5 (815–845 aa). The information of the residues that are involved in interaction with these residues and the nature of interaction have been indicated in Table 2.

**Table 2:** Residues predicted to occur at the interface of the Msh4–Msh5 heterodimer.

Protein	Residue number	Protein	Interacting residue
Msh4	H746	Msh5	K726, T728, I730
Msh4	I753	Msh5	L737
Msh4	I793	Msh5	F737
Msh4	V795	Msh5	F699, L700
Msh4	I799	Msh5	L700, A707
Msh4	P802	Msh5	L710
Msh4	I804	Msh5	L710, I740
Msh4	I811	Msh5	L736, I740
Msh4	A818	Msh5	I845
Msh4	K819	Msh5	D732
Msh5	H264	Msh4	D283
Msh5	D269	Msh4	K284
Msh5	S819	Msh4	D722
Msh5	G821	Msh4	D722
Msh5	A825	Msh4	M723
Msh5	V827	Msh4	K724
Msh5	C828	Msh4	G690,K724
Msh5	L830	Msh4	M693, A697, L700
Msh5	I834	Msh4	L700, A729, V730
Msh5	A838	Msh4	V726, A729
Msh5	L841	Msh4	F724, L728, I753
Msh5	I845	Msh4	A818

#### Design of compensatory mutations

The Msh4–Msh5 model structure can be used to predict mutational changes that are compensatory. For example, in the putative interface region, Msh4 K819, D283 and K284 are involved in ionic interactions with Msh5 D732, H264 and D269 respectively. In principle if these residues are mutated such that the overall interaction is retained, for example K819D and D732K, the phenotype is expected to be close to the wild type. The ongoing efforts are directed towards design and generation of such mutants which will further our understanding of sequence-structure-function relationship of Msh4–Msh5 complex.

### Structural interpretation of *msh4/5* mutant data

The *msh4/5* mutations are likely to cause meiotic defects by three main modes. The mutation may disrupt the structural integrity of local regions and hence affect the overall stability of the Msh4–Msh5 complex. The mutation may disrupt the interaction between the Msh4 and Msh5 proteins and prevent complex formation. Finally, the mutation may affect DNA binding by the Msh4/5 proteins.

**Figure 4:**
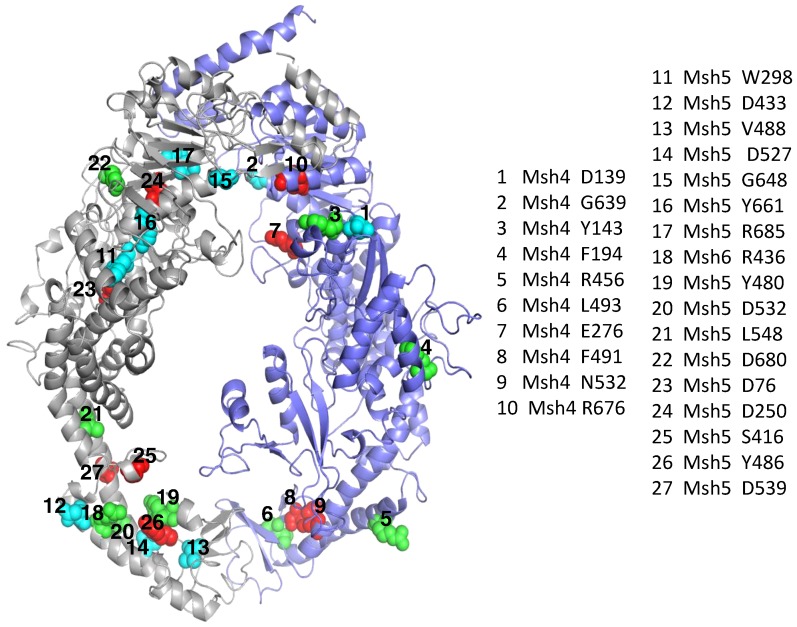
Representation of twenty seven *msh4/5* mutations on the model of the Msh4–Msh5 complex. Msh4 is coloured in violet and Msh5 is coloured in grey. Msh4/5 residues whose mutations cause null phenotype, intermediate defects in crossing over and viability or only crossover defects are represented as blue, green and red spheres respectively. 1–10 indicate Msh4 residues and 11–27 are Msh5 residues.

Twenty seven *msh4/5* mutations cause significant meiotic defects (Table S3) [6]. The position of these residues in the Msh4–Msh5 complex has been indicated in Figure 4. From the Msh4–Msh5 homology model, seventeen of these mutations are predicted to affect structural stability of the individual proteins and hence that of the overall complex (Table 3). Six *msh4/5* mutations are predicted to disrupt the interaction of the Msh4–Msh5 complex with DNA or destabilize the local structure around the DNA binding region. None of the mutations lie in the Msh4–Msh5 interface region. Meiotic defects of four *msh4/5* mutations (*msh5 D76A*, *D532A*, *D539A* and *msh4 L493A*) could not be explained with the Msh4–Msh5 modeled structure. Yeast-two-hybrid analysis suggests sixteen mutations disrupt the Msh4–Msh5 complex (Table S3) [6]. However, for *msh4 E276A*, *msh5 G648A* and *R685A* although the mutations have effects on local stability it does not affect the interaction as indicated by the yeast-two-hybrid data (Table S3). Structural explanations for meiotic defects observed in individual *msh4/5* point mutations are provided below.

**Table 3:** Classification of *msh4/5* mutations that affect protein stability or interaction with DNA.

Mutant	Mutations affecting protein stability	Mutations affecting interaction with DNA
***msh4***	*D139A*, *Y143A*, *Y194A*, *E276A*, *F491A*, *G639A*, *R676W*	*R456A*, *N532A*
***msh5***	*D250A*, *W298A*, *D433A*, *Y480A*, *D527A*, *L548A*, *G648A*, *Y661A*, *D680A*, *R685W*	*S416A*, *R436A*, *Y480A*, *V488A*

#### Null Mutations

Nine mutations, two in *MSH4* and seven in *MSH5* (*msh4 D139A*, *G639A* and *msh5 W298A*, *D433A*, *V488A*, *D527A*, *G648A*, *Y661A*, *R685W*) have meiotic defects similar to *msh4/5Δ*. In our model these involve residues stabilizing α-helical regions, residues part of the left handed α helical region of the Ramachandran map, residues involved in aromatic-aromatic interactions, cation-pi interactions, ionic interactions, hydrogen bonding and buried or solvent exposed residues. A significant proportion of interactions involve hydrogen bonding of main chain and side chain atoms or side chain and side chain atoms. A detailed information on the residues that constitute this network has been indicated in Table S3.

**Figure 5:**
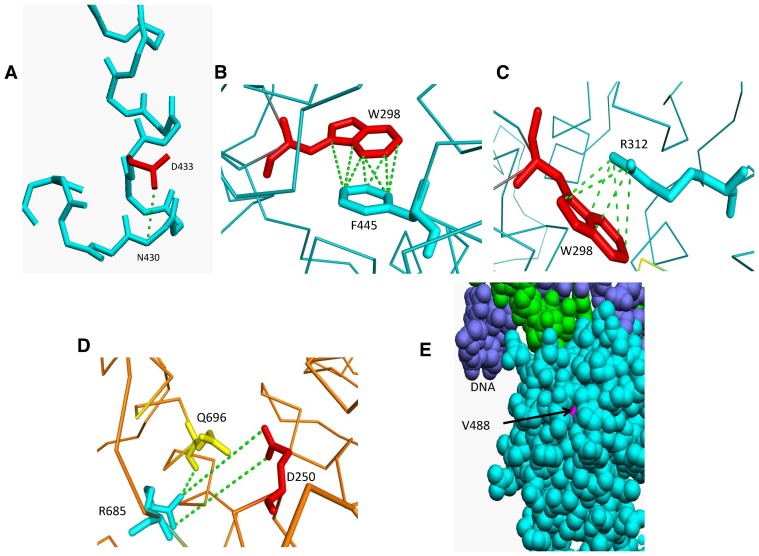
Molecular interactions of Msh4/5 residues whose mutations cause *msh4/5Δ* phenotype. A) Side chain interaction of Msh5 D433 with amide of N430. B) Aromatic-aromatic interaction between Msh5 W298 and F445. C) Cation pi interaction involving Msh5 W298 and R312. D) Ionic interaction between Msh5 R685 and D250 and hydrogen bonding between Msh5 R685 and Q696. Interactions are shown by dashed green lines. E) Tight packing of the Msh5 V488 residue.

- Residues stabilizing α-helical regions
  Msh4 D139 and Msh5 D433 are involved in stabilizing α-helical regions. The Msh4 D139 serves as an N-cap residue to stabilize a helix four residues downstream. In the case of Msh5, N430 is not a good initiator of the α-helix and hence the D433 stabilizes the structure by means of a hydrogen bond between side chain of D433 and main chain amide of N430 as shown in Figure 5A. Therefore, mutation to Alanine will disrupt these interactions thereby disrupting integrity of the local structure.
- Residues with conformations in left handed α-helical region of the Ramachandran map
  Msh5 G648 and Msh4 G639 are two residues with a positive ø dihedral angle. These angles are accommodated only in the case of Glycine due to the lack of side chain. When these residues are mutated to Alanine with this combination of ø and Ψ angles the residues experience short contacts involving their side chains and hence destabilize the structure.
- Residue involved in aromatic-aromatic and cation-pi interactions
  W298 is involved in an aromatic-aromatic interaction with F445 in Msh5 as shown in Figure 5B. In the case of *msh5 W298A*, Alanine has an aliphatic side chain which cannot participate in such an interaction and hence affects stability. The Msh5 W298 is also involved in a cation pi interaction with Msh5 R312 (Figure 5C) which will be lost when Tryptophan is mutated to Alanine.
- Residue involved in ionic interactions
  The side chain of Msh5 R685 forms a salt bridge with side chain of Msh5 D250. In addition, R685 lies before a region of insertion in Msh5. The side chain of R685 is also involved in hydrogen bonding with main chain O of Q696 as shown in Figure 5D. The residues that form a part of this insertion (T688 to Q696) have high propensity to form a β-sheet. A mutation to Alanine disrupts this network of interaction and causes destabilization.
- Residues that are buried or solvent exposed
  Msh5 V488 and Y661 are buried and also very tightly packed amongst the surrounding residues as shown for V488 in Figure 5E. This stabilization is disturbed when the residue is mutated to an Alanine as it creates a void in the region. Msh5 D527 is a solvent exposed residue. A local destabilization is caused when this is mutated to a hydrophobic residue such as Alanine.

#### Mutations with intermediate defects in crossing over and viability

In terms of severity of phenotypes, these set of mutations second the null mutations. There are nine such mutations, four in *MSH4* and five in *MSH5* (*msh4 Y143A*, *F194A*, *R456A*, *L493A* and *msh5 R436A*, *Y480A*, *D532A*, *L548A*, *D680A*). These residues are involved in aromatic-aromatic interactions, cation-pi interactions and ionic interactions. A few residues are involved in stabilizing the DNA binding region. However, in two cases *msh4 L493A* and *msh4 D532A* no explanation could be provided on the basis of the modeled structure. This is mainly due to the high sequence variation between the hMSH2–hMSH6 template and the Msh4–Msh5 model in this region.

**Figure 6:**
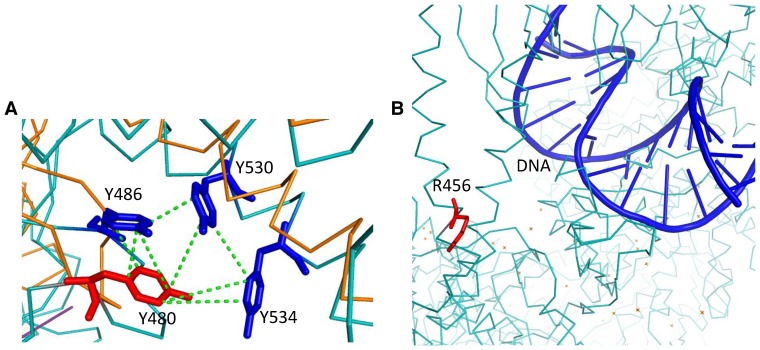
Molecular interactions of Msh4/5 residues whose mutations cause intermediate defects in crossing over and viability. A) Aromatic-aromatic interactions between Msh5 Y480, Y486, Y530 and Y534. B) Proximity of Msh4 R456 to DNA. Interactions are shown by dashed green lines.

Msh4 Y143, F194 and Msh5 Y480 are involved in aromatic-aromatic interactions with surrounding aromatic residues within a distance of 6 Å as shown for Y480 in Figure 6A. These interactions are disrupted when mutated to Alanine. Msh5 L548 is involved in tight packing which is lost when mutated to Alanine which has a smaller side chain. Msh4 R456 and Msh5 R436 are proximal to the DNA binding region as shown for R456 in Figure 6B. Hence mutation to Alanine will affect these interactions and stability of binding. The Msh5 D680 is involved in salt bridge formation with the side chains of K681 and K716 which is lost in the *msh5 D680A* mutant.

#### Mutations with only crossover defect

These mutations are deviant from the wild type only with respect to recombination frequency and have been described previously as *msh4/5-t* mutations. There are 9 such mutations distributed in both *MSH4* and *MSH5* (*msh4 E276A*, *F491A*, *N532A*, *R676W* and *msh5 D76A*, *D250A*, *S416A*, *Y486A*, *D539A*). These residues are involved in aromatic-aromatic interactions (Msh5 Y486), cation-pi interactions (Msh4 F491) and ionic interactions (Msh4 R676, Msh5 D250) with surrounding residues in 6 Å radius. Msh4 E276 and N532 are involved in a tight packing which may be disturbed upon mutation to Alanine which has a smaller side chain. The Msh5 S416 is a residue proximal to DNA and hence may affect the stability of binding. Structural explanation could not be provided for the meiotic defects observed in mutations *msh5 D76A* and *D539A*.

## Conclusions

The Msh4–Msh5 complex plays an important role in different stages of the meiotic recombination pathway. In the absence of a crystal structure for this complex, we built a homology model of the *S. cerevisiae* Msh4–Msh5 complex. The modeling studies suggest that Msh4 is most likely functionally similar to hMSH6 of the hMSH2–hMSH6 complex and likewise Msh5 is similar to hMSH2. The model also explains the functional asymmetry between Msh4 and Msh5 with respect to ATP and DNA binding mutations [6], [21]. Together these observations imply distinct roles for the Msh4 and Msh5 subunits in the recognition and binding of DNA substrates analogous to the distinct role of subunits in the MutS and MutSα complexes. The volume measurements of the cavity formed by the Msh-Msh5 complex reveals it is sufficient in size to bind an unfolded Holliday junction. The model also predicts possible interface residues and DNA binding residues whose mutations are likely to affect the function of the Msh4–Msh5 complex. *S. cerevisiae* Msh4 and Msh5 proteins have been analyzed by mutational studies [6]. The model of the Msh4–Msh5 heterodimer provides structural explanations for *msh4/5* mutations affecting crossover frequency and spore viability. The model can also facilitate the design of new mutational studies, design of structure based inhibitors of the Msh4–Msh5 complex as well as predict the functional impact of polymorphisms in the *MSH4*, *MSH5* genes. Such studies will be useful for understanding the mechanism of crossover formation by the Msh4–Msh5, Mlh1–Mlh3 pathway [11], [22], [60].

## Materials and Methods

Two possible templates (hMSH2–hMSH6 and hMSH2–hMSH3) are available for the modeling of Msh4–Msh5 complex [42], [44]. The model of the MSH4–MSH5 complex was built using the crystal structure of hMSH2–hMSH6 complex as the template (Figure S4). Since the sequence identity between the target and template is only in the order of 20%, the choice of template complex was validated using structure based alignment methods. As obtaining accurate alignment is quite difficult if the sequence similarity between the target and template is low, we used multiple algorithms for the alignment. We also considered structural environment such as solvent accessibility, secondary structures and hydrogen bonding in the manual analysis and refinement of the alignment. We started off with considering alignments obtained from fold prediction and threading algorithms PHYRE and I-TASSER [61], [62], [63]. It is well known from the CASP (Critical Assessment of protein Structure Prediction) experiments that PHYRE and I-TASSER perform quite well, in general, compared to most other methods. PHYRE generates a profile (sequence finger-print) of the family of the query sequence and secondary structures of the sequence are predicted. The profile is then searched against a fold library to choose an appropriate template onto which the sequence is threaded. I-TASSER builds models on the basis of multiple-threading alignments and iterative template fragment assembly simulations. The use of hMSH2–hMSH6 as template was validated by both servers with e-values better than 10^−10^ suggesting high confidence and that both complexes are likely to adopt the same fold. In addition, structural alignments of the *S. cerevisiae* Msh4, Msh5 protein sequences were also obtained from Bioinfo metaserver or 3D-Jury which uses the alignment information that is predicted consistently by other reliable servers [64]. We used such metaservers as they employ multiple methods and provide consensus and consistent results which are likely to be more accurate than the results from individual methods. The final alignment used for structural modeling is the result of refinement of alignments obtained from PHYRE, I-TASSER and 3D-Jury servers which were manually scrutinized for consistency with respect to conservation, secondary structure and solvent accessibility at various residue positions. The *S. cerevisiae* Msh2–Msh6 complex is also known to bind Holliday junction structures which further justifies its use as a template [65], [66].

The model of Msh4–Msh5 complex was built using MODELLER (version 9.10) auto-model program with added energy optimization steps [67]. We preferred using MODELLER over other comparative modeling methods as MODELLER can accept the sequence alignment between the template and target from the user (which is extremely important in the current modeling work) and also it has in-built sensitive approaches as described below to maximize the accuracy of structural models generated. MODELLER generates 3-D models of a protein on the basis of known 3-D structures of one or more proteins which are known to be related to the target. Structural restraints for model building are generated using the template structure(s) and expressed in terms of probability density functions. In the current work, structural models of Msh4 and Msh5 were separately generated using appropriate templates, as discussed already, with template structures available in the complexed form. This ensures that the 3-D models of Msh4 and Msh5 are generated in the bound forms. The models were superimposed on the template structures which are available in the complex form. The model of Msh4–Msh5 complex thus obtained was subjected to energy minimization using FoldX program which is one of the widely-used and highly effective programs for energy optimization [68], [69], [70]. This step further adjusts the side chain conformation by sampling various rotamers for each residue that correspond to lowest energy. Further, conformations of specific residues were manually refined to remove short contacts. The best structure was chosen on the basis of lowest energy and statistical parameters such as the DOPE Score.

Intra and inter protein interactions were identified using the PIC (Protein Interactions Calculator) server [71]. PIC has standard algorithms encoded for identifying various kinds of interactions such as hydrogen bonding, van der Waals interaction and salt bridge. As it is locally developed in our group and easily available we used it in the current work. The interface residues identified were further pruned on the basis of the hMSH4–hMSH5 interface residues [21]. The Multi-VORFFIP (MV) server was used to predict DNA binding residues [58]. MV has been used in the current work as it is the state of the art method that was shown to be highly sensitive and quite effective compared to many other methods in predicting functional residues in proteins [58]. DNA binding residues in hMSH2–hMSH6 template were also considered to map residues proximal to DNA binding site. A structure based multiple sequence alignment of Msh1, Msh2, Msh3, Msh4, Msh5 from yeast was constructed using the EXPRESSO server and formatted using the ESPript server [72], [73]. The h MSH2, hMSH3, hMSH6 sequences were used as reference since structures are available for the same. The DNA binding residues described in the literature were mapped on the alignment to obtain equivalences in Msh4 and Msh5 [42], [44]. Likewise the residues predicted to have high probability of DNA binding were also analyzed for conservation in the structure based multiple sequence alignment of human and yeast MSH proteins. Residue solvent accessibility was calculated using NACCESS program which is the most commonly used method over a long time for calculating solvent accessibility [74].

Homologues of Msh4 and Msh5 of not necessarily known 3-D structure were obtained by use of PSI-BLAST queried against the UNIPROT-SPROT database [75], [76]. Multiple sequence alignments were performed using the MAFFT-LINSI program [77]. The alignment was used to compute extent of conservation of residues mutated using entropic method with Scorecons server [78]. The homology search and alignment approaches used in the current work have been benchmarked in our group and are well-known to be highly sensitive and accurate.

Volume measurements were made using the 3V server, which is known to use a robust algorithm to determine the size of cavities. We used 3V to calculate the size of the cavity formed by the Msh4–Msh5 heterodimer and also to calculate the volume of Holliday junction DNA [79].

## Supporting Information

Figure S1 **Alignment of hMSH6, hMSH3 and Msh4 sequences.** 2o8bB corresponds to the hMSH6 from the hMSH2–hMSH6 complex and 3THY corresponds to hMSH3 from the hMSH2–hMSH3 complex. Highlighted in yellow are the deletions that are unique to hMSH3.
(PDF)

Figure S2 **Alignment of Msh4 and Msh5 amino acid sequences with the hMSH2–hMSH6 complex (PDB code 2o8b).** The alignment was used to model the Msh4–Msh5 complex structure. Msh4/5 residues whose mutations cause null phenotype, intermediate defects in crossing over and viability or only crossover defects are shown in blue, green and red boxes respectively.
(PDF)

Figure S3 **Structure based sequence alignment of Msh1–6 and hMSH2, hMSH3, hMSH6.** Highly conserved positions are highlighted in red, positions with conservative substitutions are highlighted in a blue box with residues marked in red. DNA binding residues identified from literature survey are highlighted in a yellow box. Residues predicted by Multi-VORFFIP (MV) server to have high probability of DNA binding are highlighted in a black box.
(PDF)

Figure S4 **Protocol used to model Msh4–Msh5 structure.** The sequences were submitted to three structure prediction servers and the alignment obtained from all these were compiled and manually curated to get final alignment which was provided to MODELER to build the structure. The structure was then energy minimized and checked for stereo-chemical quality (removal of short contacts).
(PDF)

Table S1 **Sequence to structure mapping for Msh4.**
(XLSX)

Table S2 **Sequence to structure mapping for Msh5.**
(XLSX)

Table S3 **Explanation for meiotic defects observed in** ***msh4/5*** **mutants based on the homology model.**
(DOCX)

Table S4 **Probability of each residue to bind/stabilize DNA.**
(XLSX)
