# Supplementary material for: Structural Insights into Saccharomyces cerevisiae Msh4–Msh5 Complex Function Using Homology Modeling
Source: PLoS One. 2013 Nov 14;8(11):e78753. doi: 10.1371/journal.pone.0078753 (PMC3828297; doi:10.1371/journal.pone.0078753)
Supplement: Figure S1 — Alignment of hMSH6, hMSH3 and Msh4 sequences. 2o8bB corresponds to the hMSH6 from the hMSH2–hMSH6 complex and 3THY corresponds to hMSH3 from the hMSH2–hMSH3 complex. Highlighted in yellow are the deletions that are unique to hMSH3. (PDF) [file pone.0078753.s001.pdf]

10 20 30 40 50

2o8bB (422) dLVICYK̃VGkFỸẼLỸHmDAliGvsẽLgLvfM̃kgnwAh̃SGFPẼiAFgrYSd̃

ββββββ βββββααααααααααα βββββ333αααααα

3THY (217) ksiyt̃plẼlq̃ỸieMk̃qqhkd̃AVLCVec̃gykYr̃FFge

ααααααααααα ββββββ βββββαα

Msh4 MSESNLSSFISTNYFNLRSAANSSNSISKPSTKKSIRNQKSPTNISSW

ααααααα βββββ αα

60 70 80 90 100

2o8bB (472) SLVq̃kgỹk̃VAR̃VẼQ̃T̃ẽt̃pem̃Mear̃C̃rkmahis̃kyd̃r̃vVr̃R̃eiCR̃IITkGT

αααα ββββββ ααααααααα 333 βββββββ

3THY (253) D̃AeiAarẽLniychldhñfmtAsIpthrLfvhVr̃rLVakgỹK̃VGVVk̃q̃t̃ẽ

αααααααα βββ ββββββββ333αααααααααα ββββββ

Msh4 ALKKKTLQIAETTWENN----------EKDSTHSHYLMTGSMASRTATSL

αααα β ββββ

110 120 130 140 150

2o8bB (522) q̃T̃ỹs̃vlẽgdps̃enys̃k̃YLLSLk̃Ẽk̃eedssh̃tR̃aỸGVC̃FVd̃T̃S̃lGk̃FfIGq

βββββββββ ββββββββ βββββ

3THY (303) t̃aalk̃aigdñrss̃lfs̃R̃kltaLỸT̃K̃S̃T̃lIGed̃VÑpliklavnvd̃eimTdt

αααα βββββββ

Msh4 SRYSTNASLLGPSIDCVLCCIYEVPRDISTR--IGLCIINCNTGQMYLSD

βββββββ

160 170 180 190 200

2o8bB (573) fs̃Dd̃rhC̃s̃r̃FrtLvahypPvQ̃VLfẽkgnLs̃keT̃ktILksslscSlqẽgli

βββ ααααααααα βββββββ ααααααα ββββββ

3THY (355) sTsỸLLCISẽnknifIGIVGVQ̃PAT̃gẽVvfD̃sfqDs̃as̃Rs̃ẽLẽt̃r̃Ms̃slq̃

βββββββ βββββββ βββββββ αααααααα

Msh4 FMDSQIYIRVVHKLQIYQPTEILIPSSSLAPTVSKLATMIKFN-------

βββ

210 220 230 240 250

2o8bB (623) pgsqFWdaskTLrtLleeẽỸFr̃ẽklsdgiVmLpqvLkgm̃tsesdsigltp

αααααααααα ααααα ββ ββ

3THY (414) PVẼLLLPs̃aLs̃eqT̃ẽaLihratsvsvqddrir̃vẽr̃md̃niỹFeyshAfq̃aV

βββββ ααααααααααααα βββββ 333 αααααααα

Msh4 ----------VAETVKIEEGSRKCFNSQDGLAAITKYLMDDTKKDLKIEE

αααααα

260 270 280 290 300

2o8bB (674) geks̃ẽLALSALGGC̃VfỹLkkCl ID̃qẼLLS̃mAnFẽeyipld̃sdt̃vskayqr
333 αααααααααααααααα ααααα βββ 3333

3THY (464) tẽfyakgivnlekPVIcSLAAIIk̃ỸLkeFnLekMLsk̃pẽñFkq̃ls̃skmef
ααα ααααααααααααααααα 333 333βββ

Msh4 IIDKTFALCAASAAISYMEEIISKSSRNLNAFRKLRIQFEGTEN-----T
ααααααααα ααααα 33

310 320 330 340 350

2o8bB (733) M̃VLd̃avT̃LnñLeIflngtñgst̃ẽgT̃LLerVd̃t̃C̃hT̃pFGkr̃lLk̃qwLC̃aPL
αααααα ααααα ααααααααααααα

3THY (526) MtIñGt̃TLr̃ñLeILq̃-ñqt̃dmkkgSLlwvLd̃hT̃kT̃s̃FGr̃r̃kLk̃k̃wVtqPl
ββ αααααα ααααα ααααααααααααα

Msh4 MLIDSKTVRGLELVENKLD-KNGISLWKFLDTTSTKMGQRSLRNSILQPL
αααααα ααααα ααααααααααααα

360 370 380 390 400

2o8bB (783) CnhyaIñdRLd̃AIẽd̃Lmvvpdk̃iseVveLLkkLpd̃Lẽr̃llSk̃IH̃ñvGSpl
ααααααααααααααα ααααααααααα ααααααααααααα αα

3THY (576) lklreInarLd̃AVs̃eVlhs̃es̃--------------------------s̃Vf
ααααααααααααααααα αα

Msh4 TDRGSIEMRLEALEELKANDDLLQKLRLEMKSLPDLDKLFSRLLCINHSA
ααααααααααααααα αα

410 420 430 440 450

2o8bB (833) k̃sqnhpd̃sr̃AiM̃ỹeẽttỸskkkIid̃Fls-ALeGFk̃vMckIigiMeẽvadg
αααα 3333 ααααααααααααααα ααααααααααααααααααα

3THY (600) gq̃Iẽñh̃Lr̃klPD̃IeR̃gLC̃S̃IyHk̃kCs̃Tq-ẼFFlIVk̃tLyh̃Lk̃sẽFq̃aIip
ααααααα ααααααααα αα αααααααααααααααααααααα

Msh4 IKPDQRINYVLLLKETLQSVKSLKDALNDQLIQSRLISETKKIFNNDAIM
αααα ααααααα αα ααααααααααααααααααα

460 470 480 490 500

2o8bB (882) FkS̃k̃iLkqVIslqtkñpẽgR̃FPdLtveLnrWd̃tafdhekArktgLiT̃pka
αααααα αααααααα αααααα

3THY (649) aVnshIqsd̃lLr̃tvIlẽIPelLspVehylkiLnẽqaAk̃vgd̃kt̃ẽLfkd̃ls̃
ααααα ααααααα ααααα αααααα αααα 33

Msh4 EIEKLINSCINEDCVWASSAIQLLNQRS--YAVKSDSNGLLDVSRQIYKE
αα α αααααα α

510 520 530 540 550

2o8bB (932) g F s̃ D y d̃ q a l A d i r ẽ n e q̃ s l l E y l ẽ K q̃ r n r̃ i g c r t I v y w G i g r n r̃ y Q L e I p

α α α α α α α α α α α α α α α α α α α α α 3 3 3 β β β 3 3 3 β β β β

3THY (699) d f p l I k̃ k r k d̃ e i q g V i d e I r m h̃ l q e I r k i L k ñ p s̃ a q Y v t v s g q ẽ F M I ẽ i k

3 α α α α α α α α α α α α α α α α α α α α α α α α α α α α β β β β β β β β β β β β

Msh4 V K E E F F R E V E D L T A K N K I N L D H N – – – – – – Y D S A R G F Y L R I K R Q E F T D D V A

α α α α α α α α α α α α α α α α α α α α β β β β β β

560 570 580 590 600

2o8bB (983) e n f t t r ñ l p ẽ y e l k s t k k g c̃ K r̃ Y w̃ t k t̃ I e k k̃ L a ñ L i n A ẽ e r r̃ d v S̃ l k̃ d C̃ M

β β β β β β β β β α α α α α α α α α α α α α α α α α α α α α α α α α

3THY (749) ñ s a v s c i p t d W v k v g s t k a v s̃ r̃ F H S̃ p f I v e n ỹ r h L ñ q l r̃ ẽ q̃ l v l d̃ C s a e W

3 3 3 β β β β β β β β β β α α α α α α α α α α α α α α α α α α α α α α α α α α

Msh4 T – – – – – L P D V F I S R T I K K N Y I E C T T L N I I K K N A R L K E V M E E I L L L S E E T V

β β β β β β β β α α α α α α α α α α α α α α α α α α α α α α α α

610 620 630 640 650

2o8bB (1034) r r l F ỹ n F d̃ k n y k d W q̃ s A V ẽ c I a v L D̃ V L L C̃ L A n y S̃ r g g d g p m̃ c r̃ P v I l l p e

α α α α α α α α α α α α α α α α α α α α α α α α α α α α α α α α β β β β

3THY (799) l d f L ẽ k F s e h ỹ h̃ s L c̃ k̃ A V h h̃ L A t V D̃ C I F S L A k̃ V A k q – – g d y c r̃ P t V q̃ e ẽ –

α α α α α α α α α α α α α α α α α α α α α α α α α α α α α α α α β β β

Msh4 D E L L D K I A T H I S E L F M I A E A V A I L D L V C S F T Y N L K E N N – – – – Y T I P I F T N

α α α α α α α α α α α α α α α α α α α α α α α α α α α α α α α α β β

660 670 680 690 700

2o8bB (1084) d t p p F L ẽ L k g S r̃ h P c g d d f i p n d I l l G c e e e e q k a y C V L V t̃ G p ñ m g G K̃ s̃ t

β β β β β β β β β β β β β β β β α α α

3THY (846) – – – R̃ k I v I k ñ G r h P l l g e q v p n n T̃ d̃ L s̃ ẽ d – – – – s̃ ẽ r̃ V M̃ I I t g p ñ m g g K̃ s s

β β β β β β β β β β β β β β β β β α α α α α

Msh4 N – – – – L L I R D S R H P L L E K V L K N F V P N T I S S T K H S S S L Q I I T G C N M S G K S V

β β β β β β β β β β β β β β β β α α α

710 720 730 740 750

2o8bB (1143) L M r Q A G L L A V M A Q̃ M G C̃ ỹ V P A e v C r̃ L T p I d̃ R̃ V F T̃ r̃ L g – – – – – – – – s t F f v E

α α α α α α α α α α α α α β β β β β β β β β β α α α α α

3THY (896) Ỹ I k Q V A L I T I M A Q I G S ỹ V P A ẽ e A t I G I V d̃ G I F T̃ r M g – – – – – – – – s t f m e ẽ

α α α α α α α α α α α α α α β β β β β β β β β β α α α α

Msh4 Y L K Q V A L I C I M A Q M G S G I P A L Y G S F P V F K R L H A R V C N D S M E L T S S N F G F E

α α α α α α α α α α α α β β β β β β β β α α α α

760 770 780 790 800

2o8bB (1194)L s ẽ T A s I L m h̃ A **t̃** a h **S̃** L V L V D̃ *ẽ* L *G* r g t̃ a t f D̃ G t A I A n A V V k e L A e t i *k* C̃ R T̃

α α α α α α α α α α β β β β β α α α α α α α α α α α α α α α α α α β β

3THY (947) l t d T a ẽ i i r k A **t̃** s q̃ S̃ L V I L d *ẽ* L *G* r̃ *g* t s t h d G i a i A ỹ a t L ẽ y F I r d v *k* S̃ L T̃

α α α α α α α α α α β β β β β α α α α α α α α α α α α α α α α α α β β

Msh4 MKEMAYFLDD I NTETLL I LDELGRGS S I ADGFCVSLAVTEHLLR−TEATV

α α α α α α α α α α β β β β β α α α α α α α α α α α α α α α α α α β β

810 820 830 840 850

2o8bB (1244)L F **S̃** **T** h̃ y h s L v ẽ **d** **y** s q n *v* a V r l G h M a ç m t f l *y* k f i k *g* − − − − − − − − − − − − − −

β β β α α α α α α β β β β β β β β β β β β β β

3THY (997) L F V **T** h̃ y p p V c̃ e L ẽ k ñ ỹ h q̃ V G ñ ỹ **h** **M** g f l v f *l* y q i **t** r̃ *g* − − − − − − − − − − − − − −

β β β α α α α α α α α β β β β β β β β β β β

Msh4 F L S THFQD I PK I MSKKPAVSHLHMDAVLLNDNSVKMNYQLTQKSVA I ENS

β β β α α α α α β β β β β β β β β β

860 870 880 890 900

2o8bB (1293)− − − a **c̆** p *k* s y G − − − − − − − F n a A r l A *n* L P e ẽ V I q k̆ G − − − − − − − h̃ r k a r̃ e f ẽ k

α α α α α α α α α α α α α α α α α α α α α

3THY (1078)− − a h k̃ s k ẽ l − − − − − − − e g l i ñ t k r̃ k̃ r l k ỹ f a k̆ l − − − − − − − − w t m h ñ a q **d̃** l

α α α α α α α α α α α α α α α α α α α α α α α α α α α α α α α

Msh4 G I RVVKK I FNPD I I AEAYN I HSLLK I AKARTENEDSNGVVDQKT I NQMKR

α α α α α α α α α α α α α α α

910 920 930

2o8bB (1326)m n q̃ s l r̃ l f r e

3THY (1111) q k w t e ẽ f n m ẽ

α α α α α α α α α α

Msh4 I HNLVA I LKECAGNEKEPLTLGKLKE I NSDF I ENFEE

Key to JOY alignments

solvent inaccessible	UPPER CASE	X
solvent accesible	lower case	x
positive ϕ	*italic*	*x*
cis−peptide	breve	x̆
hydrogen bond to other sidechain	tilde	x̃
hydrogen bond to mainchain amide	**bold**	**x**
hydrogen bond to mainchain carbonyl	underline	x
disulphide bond	cedilla	ç
