# Supplementary material for: Structural Insights into Saccharomyces cerevisiae Msh4–Msh5 Complex Function Using Homology Modeling
Source: PLoS One. 2013 Nov 14;8(11):e78753. doi: 10.1371/journal.pone.0078753 (PMC3828297; doi:10.1371/journal.pone.0078753)
Supplement: Figure S2 — Alignment of Msh4 and Msh5 amino acid sequences with the hMSH2–hMSH6 complex (PDB code 2o8b). The alignment was used to model the Msh4–Msh5 complex structure. Msh4/5 residues whose mutations cause null phenotype, intermediate defects in crossing over and viability or only crossover defects are shown in blue, green and red boxes respectively. (PDF) [file pone.0078753.s002.pdf]

```
                      10        20        30        40        50
2deB   ( 520 )

2o8bB  ( 422 ) dLVICYK̃VGkFỸẼLỸHmDAliGvsẽLgLvfM̃kgnwAh̃SGFPẼiAFgrYSd̃
                 ββββββ   ββββββααααααααααα           βββββ333αααααα
Msh4             MSESNLSSFISTNYFNLRSAANSSNSISKPSTKKSIRNQKSPTNISSW

                      60        70        80        90       100
2deB   ( 520 )                                                 gt

2o8bB  ( 472 ) SLVq̃k̃gỹk̃VAR̃VẼQ̃T̃ẽt̃pem̃Mear̃C̃rkmahis̃kyd̃r̃vVr̃R̃ẽiCR̃IITkGT
               αααα     ββββββ    ααααααααα        333   βββββββ
Msh4           ALKKKTLQIAETTWENN----------EKDSTHSHYLMTGSMASRTATSL

                     110       120       130       140       150
2deB   ( 522 ) q̃T̃ỹs̃vlẽgdpsenys̃k̃YLLSLk̃Ẽk̃eedssh̃tR̃aỸGVČFVd̃TS̃lGk̃FfIGq
                                ββββββββ       ββββββββ      βββββ
2o8bB  ( 522 ) q̃T̃ỹs̃vlẽgdps̃enys̃k̃YLLSLk̃Ẽk̃eedssh̃tR̃aỸGVČFVd̃TS̃lGk̃FfIGq
                                ββββββββ       ββββββββ      βββββ
Msh4           SRYSTNASLLGPSIDCVLCCIYEVPRDISTR--IGLCIINCNTGQMYLSD
                                ββββββββ       ββββββββ      βββββ

                     160       170       180       190       200
2deB   ( 573 ) fs̃Dd̃rhC̃s̃r̃FrtLvahypPvQ̃VLfẽkgnLs̃keT̃ktILksslscSlqẽgli
               βββ    ααααααααα  βββββββ    ααααααα      βββββ
2o8bB  ( 573 ) fs̃Dd̃rhC̃s̃r̃FrtLvahypPvQ̃VLfẽkgnLs̃keT̃ktILksslscSlqẽgli
               βββ    ααααααααα  βββββββ    ααααααα      βββββ
Msh4           FMDSQIYIRVVHKLQIYQPTEILIPSSSLAPTVSKLATMIKFN-------
               βββ    ααααααααα  βββββββ    ααααααα      βββββ

                     210       220       230       240       250
2deB   ( 623 ) pgsqFWdaskTLrtLleeẽỸFr̃ẽklsdgiVmLpqvLkgm̃tsesdsigltp
                      αααααααααα             ααααα ββ        ββ
2o8bB  ( 623 ) pgsqFWdaskTLrtLleeẽỸFr̃ẽklsdgiVmLpqvLkgm̃tsesdsigltp
                      αααααααααα             ααααα ββ        ββ
Msh4           ----------VAETVKIEEGSRKCFNSQDGLAAITKYLMDDTKKDLKIEE
                      αααααααααα             ααααα ββ        ββ
```

```
                260                 270                 280                 290                 300
2deB  (674) geks̃ẽLALSALGGC̃VfỹLkkcliD̃qẼLLS̃mAnFẽeyipld̃sdt̃vskayqr
               333αααααααααααααααααα      ααααα       βββ     3333
2o8bB (674) geks̃ẽLALSALGGC̃VfỹLkkCliD̃qẼLLS̃mAnFẽeyipld̃sdt̃vskayqr
               333αααααααααααααααααα      ααααα       βββ     3333
Msh4        IIDKTFALCAASAAISYMEEIISKSSRNLNAFRKLRIQFEGTEN-----T
               333αααααααααααααααααα      ααααα       βββ     3333

                310                 320                 330                 340                 350
2deB  (733) M̃VLd̃avT̃LnñLeIflngtñgst̃ẽgT̃LLerVd̃t̃C̃hT̃pFGkr̃lLk̃qwLC̃aPL
                 αααααα                     ααααα         ααααααααααααα
2o8bB (733) M̃VLd̃avT̃LnñLeIflngtñgst̃ẽgT̃LLerVd̃t̃C̃hT̃pFGkr̃lLk̃qwLC̃aPL
                 αααααα                     ααααα         ααααααααααααα
Msh4        MLIDSKTVRGLELVENKLD-KNGISLWKFLDTTSTKMGQRSLRNSILQPL
                 αααααα                     ααααα         ααααααααααααα

                360                 370                 380                 390                 400
2deB  (783) CnhyaIñdRLd̃AIẽd̃Lmvvpdk̃iseVveLLkkLpd̃Lẽr̃llSk̃IH̃ñvGSpl
               αααααααααααααααα  ααααααααααα       ααααααααααααα  αα
2o8bB (783) CnhyaIñdRLd̃AIẽd̃Lmvvpdk̃iseVveLLkkLpd̃Lẽr̃llSk̃IH̃ñvGSpl
               αααααααααααααααα  ααααααααααα       ααααααααααααα  αα
Msh4        TDRGSIEMRLEALEELKANDDLLQKLRLEMKSLPDLDKLFSRLLCINHSA
               αααααααααααααααα  ααααααααααα       ααααααααααααα  αα

                410                 420                 430                 440                 450
2deB  (833) k̃sqnhpdsraimyeẽttyskkkIid̃Fls-ALeGFk̃vMckIigiMeẽvadg
             αααα  3333     αααααααααααααα  αααααααααααααααααααα
2o8bB (833) k̃sqnhpd̃sr̃AiM̃yeẽttỸskkkIid̃Fls-ALeGFk̃vMckIigiMeẽvadg
             αααα  3333     αααααααααααααα  αααααααααααααααααααα
Msh4        IKPDQRINYVLLLKETLQSVKSLKDALNDQLIQSRLISETKKIFNNDAIM
             αααα  3333     αααααααααααααα  αααααααααααααααααααα

                460                 470                 480                 490                 500
2deB  (882) FkS̃k̃iLkqVIslqtkñpẽgR̃FPdLtveLnrWd̃tafdhekArktgLiT̃pka
                αααααα                      ααααααααα       αααααα
2o8bB (882) FkS̃k̃iLkqVIslqtkñpẽgR̃FPdLtveLnrWd̃tafdhekArktgLiT̃pka
                 αααααα                      ααααααααα       αααααα
Msh4        EIEKLINSCINEDCVWASSAIQLLNQRS--YAVKSDSNGLLDVSRQIYKE
                 αααααα                      ααααααααα       αααααα
```

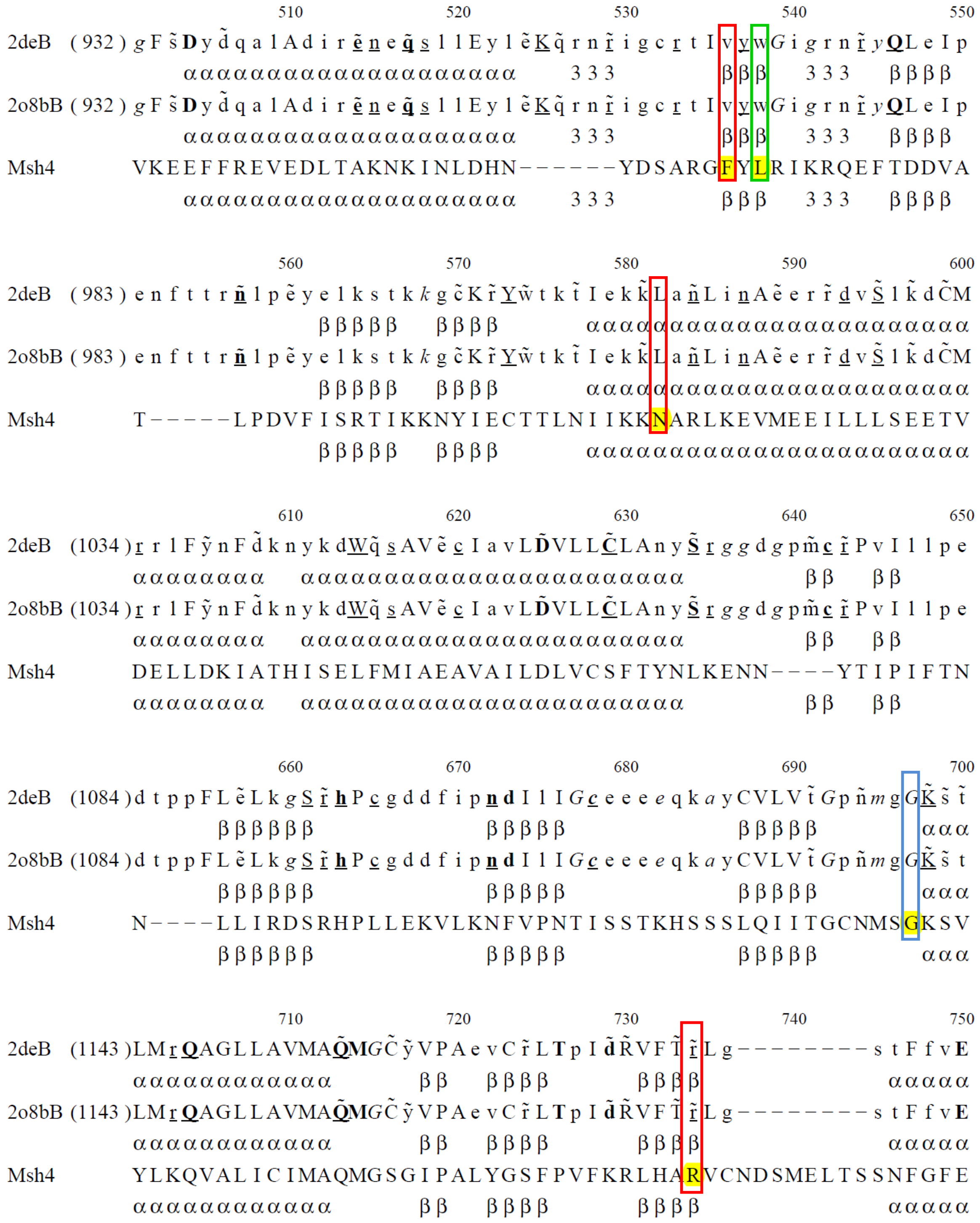

510 520 530 540 550
2deB (932) gFs̃Dyd̃qalAdirẽneq̃sllEyleKq̃rnr̃igcrtIvywGigrnr̃yQLeIp
ααααααααααααααααααααα 333 βββ 333 ββββ
2o8bB (932) gFs̃Dyd̃qalAdirẽneq̃sllEyleKq̃rnr̃igcrtIvywGigrnr̃yQLeIp
ααααααααααααααααααααα 333 βββ 333 ββββ
Msh4 VKEEFFREVEDLTAKNKINLDHN------YDSARGFYLRIKRQEFTDDVA
ααααααααααααααααααααα 333 βββ 333 ββββ
560 570 580 590 600
2deB (983) enftttrñlpẽyelkstkkgc̃Kr̃Yw̃tkt̃Iekk̃LañLinAẽerr̃dvS̃lk̃dC̃M
ββββββ ββββ αααααααααααααααααααααααα
2o8bB (983) enftttrñlpẽyelkstkkgc̃Kr̃Yw̃tkt̃Iekk̃LañLinAẽerr̃dvS̃lk̃dC̃M
ββββββ ββββ αααααααααααααααααααααααα
Msh4 T-----LPDVFISRTIKKNYIECTTLNIIKKNARLKEVMEEILLLSEETV
ββββββ ββββ αααααααααααααααααααααααα
610 620 630 640 650
2deB (1034) rrlFỹnFd̃knykdWq̃sAVẽcIavLD̃VLLC̃LAnyS̃rggdgpm̃cr̃PvIllpe
ααααααααα ααααααααααααααααααααααααα ββ ββ
2o8bB (1034) rrlFỹnFd̃knykdWq̃sAVẽcIavLD̃VLLC̃LAnyS̃rggdgpm̃cr̃PvIllpe
ααααααααα ααααααααααααααααααααααααα ββ ββ
Msh4 DELLDKIATHISELFMIAEAVAILDLVCSFTYNLKENN----YTIPIFTN
ααααααααα ααααααααααααααααααααααααα ββ ββ
660 670 680 690 700
2deB (1084) dtppFLẽLkgSr̃hPcgddfipndIlIGceeeeqkayCVLVt̃Gpñmg GK̃s̃t̃
ββββββ ββββββ βββββ ααα
2o8bB (1084) dtppFLẽLkgSr̃hPcgddfipndIlIGceeeeqkayCVLVt̃Gpñmg GK̃s̃t
ββββββ ββββββ βββββ ααα
Msh4 N----LLIRDSRHPLLEKVLKNFVPNTISSTKHSSSLQIITGCNMSGKSV
ββββββ ββββββ βββββ ααα
710 720 730 740 750
2deB (1143) LMrQAGLLAVMAQ̃MGC̃ỹVPAevCr̃LTpId̃R̃VFT̃r̃Lg---------stFfvE
αααααααααααα ββ ββββ ββββ ααααα
2o8bB (1143) LMrQAGLLAVMAQ̃MGC̃ỹVPAevCr̃LTpId̃R̃VFT̃r̃Lg---------stFfvE
αααααααααααα ββ ββββ ββββ ααααα
Msh4 YLKQVALICIMAQMGSGIPALYGSFPVFKRLHARVCNDSMELTSSNFGFE
αααααααααααα ββ ββββ ββββ ααααα

760 770 780 790 800

2deB (1194)L s ẽ T A s I L m h̃ A t̃ a h S̃ L V L V D̃ ẽ L G r g t̃ a t f D̃ G t A I A n A V V k e L A e t i k C̃ R T̃

αααααααααα βββββ αααααααααααααααααα ββ

2o8bB (1194)L s ẽ T A s I L m h̃ A t̃ a h S̃ L V L V D̃ ẽ L G r g t̃ a t f D̃ G t A I A n A V V k e L A e t i k C̃ R T̃

αααααααααα βββββ αααααααααααααααααα ββ

Msh4 MKEMAYFLDDINTETLLILDELGRGSSIADGFCVSLAVTEHLLR−TEATV

αααααααααα βββββ αααααααααααααααααα ββ

810 820 830 840 850

2deB (1244)L F S̃ T h̃ y h s L v ẽ d y s q n v a V r l G h M a c m t f l y k f i k g−−−−−−−−−−−−−−

βββ αααααα ββββββββ ββββββ

2o8bB (1244)L F S̃ T h̃ y h s L v ẽ d y s q n v a V r l G h M a c m t f l y k f i k g−−−−−−−−−−−−−−

βββ αααααα ββββββββ ββββββ

Msh4 FLSTHFQDIPKIMSKKPAVSHLHMDAVLLNDNSVKMNYQLTQKSVAIENS

βββ αααααα ββββββββ ββββββ

860 870 880 890 900

2deB (1293)−−−a c̃ p k s y G−−−−−−−F n a A r l A n L P e ẽ V I q k̃ G−−−−−−−h̃ r k a r̃ e f ẽ k

αα αααααα ααααααα αααααα

2o8bB (1293)−−−a c̃ p k s y G−−−−−−−F n a A r l A n L P e ẽ V I q k̃ G−−−−−−−h̃ r k a r̃ e f ẽ k

αα αααααα ααααααα αααααα

Msh4 GIRVVKKIFNPDIIAEAYNIHSLLKIAKARTENEDSNGVVDQKTINQMKR

αα αααααα ααααααα αααααα

910 920 930

2deB (1326)m n q̃ s l r̃ l f r e

2o8bB (1326)m n q̃ s l r̃ l f r e

Msh4 IHNLVAILKECAGNEKEPLTLGKLKEINSDFIENFEE

```
                          10                  20                  30                  40                  50
2deA   ( 126 )

2o8bA (   1 )  m̃avq̃pket̃lq̃lesaaẽvgFvrffqgMpek̃pttTVr̃LFdr̃gdfyT̃AH̃geD̃A
                           αααααααααααα                      ββββββ          ββββαααα
Msh5


                          60                  70                  80                  90                 100
2deA   ( 126 )

2o8bA (  51 )  llAArevFktqgvIkym̃Gpagak̃nlqS̃VvLs̃kmÑFesFVkdLLlvr̃qỹrV
               αααααα              βββ              ββββββ  αααααααααααααα     ββ
Msh5            MSHEWLISASETMRSIGNGEGLRDKGAVVANNDGE-------------FN


                          110                 120                 130                 140                 150
2deA   ( 126 )                                          gñls̃qfedilfgnñigVVGVkmsavdgq
                                                           333               βββββ
2o8bA ( 101 )  ẽVỹknragskeñdW̃ylaykASPGñls̃qFediLfgnñigVVGVkmsavdgq
               βββββ           βββββββ         333               βββββ
Msh5           EGDTNREEDSSTIFSFDFDEEIVMCIDFSGGKLGCSILDYHTKTLKAFDQ
                                                           333               βββββ

                          160                 170                 180                 190                 200
2deA   ( 159 )  r̃qVGVGyVdsiq̃r̃klGLcẽFpDñd̃qFs̃nLeALLiQigPkẽCVLpGgẽtag
                 ββββββββ         ββββββββ          αααααααααα       ββββ
2o8bA ( 159 )  r̃qVGVGyVdsiq̃r̃klGLcẽFpDñd̃qFs̃nLeALLiQigPkẼCVLpGgẽtag
                 ββββββββ         ββββββββ          αααααααααα       ββββ
Msh5           DYVVNKTTISSHDLIDDADMSSNDISLLLGLLIMEANPTVCLVPARLEDW
                 ββββββββ         ββββββββ          αααααααααα       ββββ

                          210                 220                 230                 240                 250
2deA   ( 209 )  d̃mgkLr̃qIIqrggilit̃erkkadfstKd̃iyq̃d̃lN-r̃gkkgẽqmns̃avlp-
               αααααααααα        ββββ                αααα                        3
2o8bA ( 209 )  d̃mgkLr̃qIIqrggilit̃erkkadfstKd̃iyq̃d̃lN-r̃gkkgẽqmns̃avlp-
               αααααααααα        ββββ                αααα                        3
Msh5            IFDYIKTKCDEINCRLELQPIKRFKKWDLLQSLQLRGHDNQTILNDILSN
               αααααααααα        ββββ                αααα                        3
```

```
                   260        270        280        290        300
2deA  ( 260 ) −emeñqvAvsSlSAVikflellsdd−−−−−−snfgq̃Feltt−−−−−−−−−
                333  αααααααααααααα
2o8bA ( 260 ) −emeñqvAvsSlSAVikFlellsdd−−−−−−snfgq̃Feltt−−−−−−−−−
                333  αααααααααααααα
Msh5          SKFTTTVTLGTVGCILANHEQLGEYNDSTASSNMVTGRLVQNAFEDVIHG
                333  αααααααααααααα

                   310        320        330        340        350
2deA  ( 294 ) −−−fd̃fs̃qỹMkLd̃iaAvrALnLfqgs−−−−−−−−−−tgsqsLaalLñkC̃k
                          ββ αααααα                      ααααα
2o8bA ( 294 ) −−−fd̃fs̃qỹMkLd̃iaAvrALnLfqgs−−−−−−−−−−tgsqsLaalLñkC̃k
                          ββ αααααα                      ααααα
Msh5          IRYIDIRDRMVLDENTISALHIFPTAHKLGHDKMMRNGFFSVFELFNQVS
                          ββ αααααα                      ααααα

                   360        370        380        390        400
2deA  ( 335 ) T̃pQ̃Gqr̃lVñq̃wIkq̃PlmdknrIeerLnlVẽaFveD−aeLr̃qtLq̃edlLrr̃
               ααααααααααααα    ααααααααααααααααααα  ααααααα    333
2o8bA ( 335 ) T̃pQ̃Gqr̃lVñq̃wIkq̃PlmdknrIeerLnlVẽaFveD−aeLr̃qtLq̃edlLrr̃
               ααααααααααααα    ααααααααααααααααααα  ααααααα    333
Msh5          SDYARRILKSWLINPLTNKKRIETRYSIIRTLLDKQNAIIFSDLSQSIKR
               ααααααααααααα    ααααααααααααααααααα  ααααααα    333

                   410        420        430        440        450
2deA  ( 384 ) FpD̃Lñr̃LAkkFq̃rq̃aAnLqdC̃ỹrLỹq̃GInq̃LpnViqaLekhegkhqkllL
                 αααααααααα    ααααααααααααα   ααααααα
2o8bA ( 384 ) FpD̃Lñr̃LAkkFq̃rq̃aAnLqdC̃ỹrLỹq̃GInq̃LpnViqaLekhegkhqkllL
                 αααααααααα    ααααααααααααα   ααααααα
Msh5          CPDAFGFINQLRSGKSTLGTWSKVASFLEKGIAIFQLVSSLKLGSDEANI
                 αααααααααα    ααααααααααααα   ααααααα

                   460        470        480        490        500
2deA  ( 434 ) avFvtpLtdlrsdFskFqemIẽtTLd̃mdq̃Ven−heflVkpsfdpnLselr
                 ααααααααααα ααααααααααα  αααα                ααααααα
2o8bA ( 434 ) avFvtpLtdlrsdFskFqemIẽtTLd̃mdq̃Ven−heflVkpsfdpnLselr
                 ααααααααααα ααααααααααα  αααα                ααααααα
Msh5          LHDIKNKVDIS−ALKECLRKVETVIDFDTSRDTKTLTINTGVDNRLDECR
                 ααααααααααα ααααααααααα  αααα                ααααααα
```

```
                          510                 520                 530                 540                 550
2deA  (483) e i m ñ - - - - - - - d l e k̃ k M q s t l i s A A r d L g l d̃ p g k q̃ I k L d s - - - - s a q f g ỹ
            α α α α               α α α α α α α α α α α α α α α α α                   β         β       β β
2o8bA (483) e i m ñ - - - - - - - d l e k̃ k M q s t l i s A A r d L g l d̃ p g k q̃ I k L d s - - - - s a q f g ỹ
            α α α α               α α α α α α α α α α α α α α α α α                   β         β       β β
Msh5        N I Y N H L E G I L L D V A R E T Q I F L L N T M P Q E D C K T T K S L E K L V N A V Y I P Q L G Y
            α α α α               α α α α α α α α α α α α α α α α α                   β         β       β β

                          560                 570                 580                 590                 600
2deA  (522) Y F r V t̃ c k ẽ e k̃ V l r n n k n F s t v d I q̃ k n G - - V K̃ F t n s k L t s l n e e Ỹ t k n k t e
              β β β                           β β β β β β β     β     β β β β β α α α α α α α α α α α α α α α α α
2o8bA (522) Y F r V t̃ c k ẽ e k̃ V l r n n k n F s t v d I q̃ k n G - - V K̃ F t n s k L t s l n e e Ỹ t k n k t e
              β β β                           β β β β β β β     β     β β β β β α α α α α α α α α α α α α α α α α
Msh5        L V T I S V L M E P L L D G I P N L Q W E E I F R S S E N I Y F K N G R V L E L D E T Y G D I Y G A
              β β β                           β β β β β β β     β     β β β β β α α α α α α α α α α α α α α α α α

                          610                 620                 630                 640                 650
2deA  (570) y e e a q d a i v k e I v ñ i S̃ s g y v e p M q̃ t L Ñ d̃ v L A q L D̃ A V V S̃ F A h̃ v S̃ ñ g a p v p Y
            α α α α α α α α α α α α α α α α α 3 3 3 α α α α α α α α α α α α α α α α α α α α α α α α α α α α α
2o8bA (570) y e e a q d a i v k e I v ñ i S̃ s g y v e p M q̃ t L Ñ d̃ v L A q L D̃ A V V S̃ F A h̃ v S̃ ñ g a p v p Y
            α α α α α α α α α α α α α α α α α 3 3 3 α α α α α α α α α α α α α α α α α α α α α α α α α α α α α
Msh5        I S D F E I E I L F S L Q E Q I L R R K T Q L T A Y N I L L S E L E I L L S F A Q V S - - A E R N Y
            α α α α α α α α α α α α α α α α α 3 3 3 α α α α α α α α α α α α α α α α α α α α α α α α α α α α α

                          660                 670                 680                 690                 700
2deA  (620) v r̃ P a I l e k̃ G q g r I i L k a S̃ R̃ h A C v Ẽ v q d - - - - - - - - - e i a F i p n d V ỹ F ẽ k d
                β β β           β β β β β β β                                              β β β β β β
2o8bA (620) v r̃ P a I l e k̃ G q g r I i L k a S̃ R̃ h A C v Ẽ v q d - - - - - - - - - e i a F i p n d V ỹ F ẽ k d
                β β β           β β β β β β β                                              β β β β β β
Msh5        A E P Q L V E D E C I L E I I N G R H A L Y E T F L D N Y I P N S T M I D G G L F S E L S W C E Q N
                β β β           β β β β β β β                                              β β β β β β

                          710                 720                 730                 740                 750
2deA  (661) k q̃ m F H I I t g p n m G G k s̃ t Y I r̃ Q̃ T̃ G V I V L M A Q I G C F V P C e s A e V S I V d̃ C I l A
                β β β β β             α α α α α α α α α α α α α α α α α             β β β β β       β β β β
2o8bA (661) k q̃ m F H I I t g p n m G G k s̃ t Y I r̃ Q̃ T̃ G V I V L M A Q I G C F V P C e s A e V S I V d̃ C I l A
                β β β β β             α α α α α α α α α α α α α α α α α             β β β β β       β β β β
Msh5        K G R I I V V T G A N A S G K S V Y L T Q N G L I V Y L A Q I G C F V P A E R A R I G I A D K I L T
                β β β β β             α α α α α α α α α α α α α α α α α             β β β β β       β β β β
```

```
                    760       770       780       790       800
2deA   (711) rvg---------stFmaeMlẽTAsILrs̃Atkd̃SLIIId̃ẽLGr̃gtstyDGf
             ββ           αααααααααααααα      βββββ       ααααα
2o8bA  (711) rvg---------stFmaeMlẽTAsILrs̃Atkd̃SLIIId̃ẽLGr̃gtstyDGf
             ββ           αααααααααααααα      βββββ       ααααα
Msh5         RIRTQETVYKTQSSFLLDSQQMAKSLSLATEKSLILIDEYGKGTDILDGP
             ββ           αααααααααααααα      βββββ       ααααα

                    810       820       830       840       850
2deA   (761) GLAwAISeyIAt-kigAFCM̃FAThFheLt-------------------aL
             αααααααααααα     βββββ                           α
2o8bA  (761) GLAwAISeyIAt-kigAFCM̃FAThFheLt-------------------aL
             αααααααααααα     βββββ                           α
Msh5         SLFGSIMLNMSKSEKCPRIIACTHFHELFNENVLTENIKGIKHYCTDILI
             αααααααααααα     βββββ                           α

                    860       870       880       890       900
2deA   (791) anqiptVñnlhVtAltteetLtmlyqVkkgvcdqsFgihvAelAnFpkhV
             ααα   ββββββββββ    βββββββββ       ααααααα    ααα
2o8bA  (791) anqiptVñnlhVtAltteetLtmlyqVkkgvcdqsFgihvAelAnFpkhV
             ααα   ββββββββββ    βββββββββ       ααααααα    ααα
Msh5         SQKYNLLETAHVGEDHESEGITFLFKVKEGISKQSFGIYCAKVCGLSRDI
             ααα   ββββββββββ    βββββββββ       ααααααα    ααα

                    910       920       930       940       950
2deA   (841) ie------------------cAkqk̃alẽleeFq
             αα                  αααααααααα
2o8bA  (841) ie------------------cAkqk̃alẽleeFq
             αα                  αααααααααα
Msh5         VERAEELSRMINRGDDVVQQCGNLTEKEMREFQKNQEIVKKFLSWDLDLE
             αα                  αααααααααα

                    960
2deA   (   )

2o8bA  (   )

Msh5         TTTTSENLRLKLKNFLR
```

Key to JOY alignments

solvent inaccessible	UPPER CASE	X
solvent accesible	lower case	x
positive ϕ	*italic*	*x*
cis−peptide	breve	x̆
hydrogen bond to other sidechain	tilde	x̃
hydrogen bond to mainchain amide	**bold**	**x**
hydrogen bond to mainchain carbonyl	<u>underline</u>	<u>x</u>
disulphide bond	cedilla	ç
