# Supplementary material for: Structural Insights into Saccharomyces cerevisiae Msh4–Msh5 Complex Function Using Homology Modeling
Source: PLoS One. 2013 Nov 14;8(11):e78753. doi: 10.1371/journal.pone.0078753 (PMC3828297; doi:10.1371/journal.pone.0078753)
Supplement: Figure S3 — Structure based sequence alignment of Msh1–6 and hMSH2, hMSH3, hMSH6. Highly conserved positions are highlighted in red, positions with conservative substitutions are highlighted in a blue box with residues marked in red. DNA binding residues identified from literature survey are highlighted in a yellow box. Residues predicted by Multi-VORFFIP (MV) server to have high probability of DNA binding are highlighted in a black box. (PDF) [file pone.0078753.s003.pdf]

```
2o8bA_MSH2  ......................................................................
            1        10
2o8bA_MSH2  MAVQPK........ETLQLESAA...............................................
Msh1_YEAST  MKHFFRLPTAFRPISRVSLRYSSTDTA....QPKISKL................................
Msh2_YEAST  MSSTRP................................................................
MSH3_HUMAN  GP....................................................................
Msh3_YEAST  MAGQPTI.SRF...........................FKKAVKSELTHQEQEV.............AVG
Msh4_YEAST  MSESNLS.SFI.STNYFNLRSAANSSN...........................................
Msh5_YEAST  MSHEWLI.SAS.E...TMRSIG................................................
MSH6_HUMAN  MGSAPQN.SES.Q...AHVSGG................................................
Msh6_YEAST  MAPATEK.TSK.T...AHFENGSTSSQKKMKQSSLLSFFSKQVPSGTPSKKVQKPTPATLENTATDKITK

2o8bA_MSH2  ......................................................................
                      20
2o8bA_MSH2  .......EVGFVR.........................................................
Msh1_YEAST  .......KISFNKISESNS....................EKKDNLGSIDTRNCLSTQ.QDDK........
Msh2_YEAST  .......ELKFSDVSEERN...................................................
MSH3_HUMAN  ..K...................................................................
Msh3_YEAST  NGAG..SESICLDTDEEDNLSSVASTTVTNDSFPLKGS.VSSKNSKNSEKTSGTSTTFNDIDFAKKLDR.
Msh4_YEAST  ............SISKPST...................................................
Msh5_YEAST  NGEG..................................................................
MSH6_HUMAN  ..GD..................................................................
Msh6_YEAST  NPQGGKTGKLFVDVDEDNDLTIAEETVSTVRSDIMHSQEPQSDTMLNSNTTEPKSTT.TDEDLSSSQSRR

                                                α1
2o8bA_MSH2  ......................................................................

2o8bA_MSH2  ......................................................................
Msh1_YEAST  ......................................................................
Msh2_YEAST  ......................................................................
MSH3_HUMAN  ......................SA..............................................
Msh3_YEAST  IMKRR....................SDENV........................................
Msh4_YEAST  ......................................................................
Msh5_YEAST  ......................LRDKGAVV........................................
MSH6_HUMAN  ......................DSSRPTVW........................................
Msh6_YEAST  NHKRRVNYAESDDDDSDTTFTAKRKKGKVVDSESDEDEYLPDKNDGDEDDDIADDKEDIKGELAEDSGDD

2o8bA_MSH2  ......................................................................

2o8bA_MSH2  ......................................................................
Msh1_YEAST  ......................................................................
Msh2_YEAST  ......................................................................
MSH3_HUMAN  ......................................................................
Msh3_YEAST  ......................................................................
Msh4_YEAST  ......................................................................
Msh5_YEAST  ....................................................................AN
MSH6_HUMAN  ....................................................................YH
Msh6_YEAST  DDLISLAETTSKKKFSYNTSHSSSPFTRNISRDNSKKKSRPNQAPSRSYNPSHSQPSATSKSSKFNKQNE

2o8bA_MSH2  ...................................................................TT..
                                                                  30
2o8bA_MSH2  ....................................................FFQ...GMPEKPTTTV..
Msh1_YEAST  ............................LSSTEPSK.........A..SLPPSLQYVRDLMDLYKDHV..
Msh2_YEAST  ....................................................FYKKYTGLPKKPLKTI..
MSH3_HUMAN  ...............N................KRSK.........S..IYTPLELQYIEMKQQHKDAVL.
Msh3_YEAST  ......................EAEDDEEEGEEDFVKKKARKSPTA..KLTPLDKQVKDLKMHHRDKVL.
Msh4_YEAST  ...............................KKSIR.........N..QKSPTNISSWALKKKTLQIAET
Msh5_YEAST  NDGEFNEGDTNREEDSSTIFSFDFDEEIVMCIDFSG.........GKLGCSILDYHTKTLKAFDQDYVV.
MSH6_HUMAN  ETLEWLKEEKRRDEHRRRPDHPDFDASTLYVPEDFL.........N..SCTPGMRKWWQIKSQNFDLVI.
Msh6_YEAST  ERYQWLVDE..RDAQRRPKSDPEYDPRTLYIPSSAW.........N..KFTPFEKQYWEIKSKMWDCIV.
```

```
                β1          β2        α2                    β3                                  β4
2o8bA_MSH2 ....→.→      ──→ ℓℓℓℓℓℓℓℓℓℓ     TT ─..→  TT                               ──→
                      40        50        60        70                                  80
2o8bA_MSH2 ....RL.FDRGDFYTAHGEDALLAAREVFKTQGVIK..YMGPAGA.....K...........NLQSVVL
Msh1_YEAST ....VL.TQMGSFYELYFEQAIRYAPELN.ISLT........NR.AYSHG...........KVPFAGF
Msh2_YEAST ....RL.VDKGDYYTVIGSDAIFVADSVYHTQSVLKNCQLDPVTA.KNFHE...........PTKYVTV
MSH3_HUMAN .....C.VECGYKYRFFGEDAEIAARELN.IYC..HLDH......................NFMTASI
Msh3_YEAST .....V.IRVGYKYKCFAEDAVTVSRILH.IKLVPGKLTIDESNPQDCNHR...........QFAYCSF
Msh4_YEAST TWENNEKDSTHSHYLMTGSMASRTAT........................................
Msh5_YEAST .....N.KTTISSHD.LIDDADMSSN..D.ISLLLGLLI.MEAN.....PTVCLVPARLEDWIFDYIKT
MSH6_HUMAN .....C.YKVGKFYELYHMDALIGVSELG.LVFMKG......................NWAHSGF
Msh6_YEAST .....F.FKKGKFFELYEKDALLANALFD.LKIAGGRA.....................NMQLAGI

                 α3                 β5                                    β6
2o8bA_MSH2 ℓℓℓℓℓℓℓℓℓℓℓℓℓℓ      ──→                                   ─.....──→ T
                 90        100       110                                120
2o8bA_MSH2 SKMNFESFVKDLLLVRQYRVEVYKNRA......GN...KA...........SKENDWY.....LAYKASP
Msh1_YEAST PVHQLSRHLKMLVNNCGYSVTIAEQFK......KK...DVA.....DN..EANKFYRR.....VTRIVTP
Msh2_YEAST SLQVLATLLKLCLLDLGYKVEIYDKG..............................WK.....LIKSASP
MSH3_HUMAN PTHRLFVHVRRLVA.KGYKVGVVKQTE......TAALKAIG.....DN..RSSLFSRK.....LTALYTK
Msh3_YEAST PDVRLNVHLERLVH.HNLKVAVVEQAE......TSAIKKHD...PGAS..KSSVFERK.....ISNVFTK
Msh4_YEAST ..........................................................S.....LSRYSTN
Msh5_YEAST KCDEI.........N.CRLELQPIKRFKKWDLLQSLQLRGHD.....NQ....TILNDILSNSKFTTTVTL
MSH6_HUMAN PEIAFGRYSDSLVQ.KGYKVARVEQTE......TPEMMEARCRKMAHISKYDRVVRRE.....ICRIITK
Msh6_YEAST PEMSFEYWAAQFIQ.MGYKVAKVDQRE......SMLAKEMR.....EG..SKGIVKRE.....LQCILTS

               η1                                 β7                          β8
2o8bA_MSH2 T  ℓℓℓ                                  ──→                          ───
             130                    140        150                     160
2o8bA_MSH2 GNLSQFEDIL..............FGNNDMSASIGVVGVKM....S.........A.VD.GQRQVGVGY
Msh1_YEAST GTFIDEAFEN..............LRENTY...LLNI.EFPENCMS.........QVAD.TSLKVGICW
Msh2_YEAST GNIEQVNELM..............NMNIDSSIIASLKVQW....N.........S.QD.GNCIIGVAF
MSH3_HUMAN STLIGEDVNPLIKLDDAVNVDEIMTDTSTSY...LLCI.SENKENVR........DKKK.GNIFIGIVG
Msh3_YEAST ATFGVNS.....................TF...VLRG.KRILGDTNSIWAL..SRDVHQGKVAKYSLIS
Msh4_YEAST ASLLGPS..................IDCV...LCCI.YEV............P.RD.ISTRIGLCI
Msh5_YEAST GTV........................G.C.ILAN.HEQLGEYND...............STAS
MSH6_HUMAN GTQTYSVLEG.........DPS.ENYSKY...LLSL.KEKEED............SSGHTRAYGVCF
Msh6_YEAST GTLTDGDMLH..............SDLATF...CLAI.REEPGNFYNETQLDSSTIVQKLNTKIFGAAF

                    β9              α4           β10                   α5
2o8bA_MSH2 →      ────→        ℓℓℓℓℓℓℓℓℓℓℓ     ──→            ℓℓℓ.........ℓℓℓ....ℓℓℓ
             170       180       190       200       210
2o8bA_MSH2 VDSIQRKLGLCEFPDNDQF.SNLEALLIQIGPKECVLPG...GETAG.DMG.........KLR....QII
Msh1_YEAST CDVSTGEIFVQQV....YL.RDLVSAITRIQPKEILLDE...RLLEF.HIESGTWYPELVELKKFIKYQ
Msh2_YEAST IDTTAYKVGMLDIVDNEVY.SNLESFLIQLGVKECLVQDLTSNSNSNAEMQ.........KVI....NVI
MSH3_HUMAN VQPATGEVVFDSFQDSASR.SELETRMSSLQPVELLLPS...ALSE.QTE.........AL....IHRA
Msh3_YEAST VNLNNGEVVYDEFEEPNLADEKLQIRIKYLQPIEVLVNT...DDLPL.HVA.........KF....FKDI
Msh4_YEAST INCNTGQMYLSDFMDSQIY.IRVVHKLQIYQPTEILIPS...SSLAP.TVS.........KLAT.MIKFN
Msh5_YEAST SNMVTGRLVQNAFEDVIH.....................................................
MSH6_HUMAN VDTSLGKFFIGQFSDDRHC.SRFRTLVAHYPPVQVLFEK...GNLSK.ETK.........TI....LKSS
Msh6_YEAST IDTATGELQMLEFEDDSEC.TKLDTLMSQVRPMEVVMER...NNLST.LAN.........KI....VKFN

               β11                                      α6                η2
2o8bA_MSH2 ℓ     ──→       TT                              ℓℓℓℓ               ℓℓℓℓ
            220       230                              240       250       260
2o8bA_MSH2 QRGGILITE.RKK.ADF.STKD.....................IYQDLNRLLKGKKGEQMNSA.VLPEME
Msh1_YEAST KMPSQH.....RTIESF.Y.......................GLFNL....GGKEATERQLKI.QFQTF
Msh2_YEAST DRCGCVVTL.LKN.SEF.SEKD.....................VELDLTKLL....GDDL..ALSLPQKY
MSH3_HUMAN TSVS.VQDDRIR.VERM..........DNIYFEYS......HAFQAVTEFYA..KDT.VDIK.GSQIIS
Msh3_YEAST SCP..LI.....H.KQEY.DLED.....................HVVQA.................IK....VMN
Msh4_YEAST VAETVKIEE.GSR.KCF.NSQD.....................GLAAITKYLM..DDTKKDLKI.EEIID
Msh5_YEAST .....................................................................
MSH6_HUMAN LSCS.LQEGLIP.GSQFWDASKTLRTLLEEEYFREKLSDGIGVMLPQV.............LK.GMTSES
Msh6_YEAST SAPNAIFNEVKA.GEEFYDCDKTYAEIISSEYFSTE......EDWPEV.............LK.SYYDTG

                          α7                                                  β12
2o8bA_MSH2           ℓℓℓℓℓℓℓℓℓℓℓℓℓℓℓℓℓ  TT  TT                          TT    ─
                          270       280       290                          300
2o8bA_MSH2 ..........NQVAVSSLSAVIKFLELLSDDS..NFGQFELT.................TF.DFSQYMK
Msh1_YEAST ..........TQKELAALRNTLIYVSNHLPDF..SI.NFQIP.................QRQLATAIMQ
Msh2_YEAST ..........SKLSMGACNALIGYLQLLSEQD..QVGKYELV.................EH.KLKEFMK
MSH3_HUMAN GIVN.....LEKPVICSLAAIIKYLKEFNL..EKMLSKPENF.................KQLSSKMEFMT
Msh3_YEAST EKIQL.....SPSLIRLVSKLYSHMVEYNN..EQVMLIPS.................IYSPFASKIHML
Msh4_YEAST ..........KTFALCAASAAISYMEEIISKSSRNLNAFRKL.................RIQFEGTENTML
Msh5_YEAST ......................................................GIRYIDIRDRMV
MSH6_HUMAN DSIGLTPGEKSELALSALGGCVFYLKKCLI..DQELLSMANFEEYIPLDSDVTSTTRSGAIFTKAYQRMV
Msh6_YEAST ..........KKVGFSAFGGLLYYLKWLKL..DKNLISMKNIK.................EYDFVKSQHSMV
```

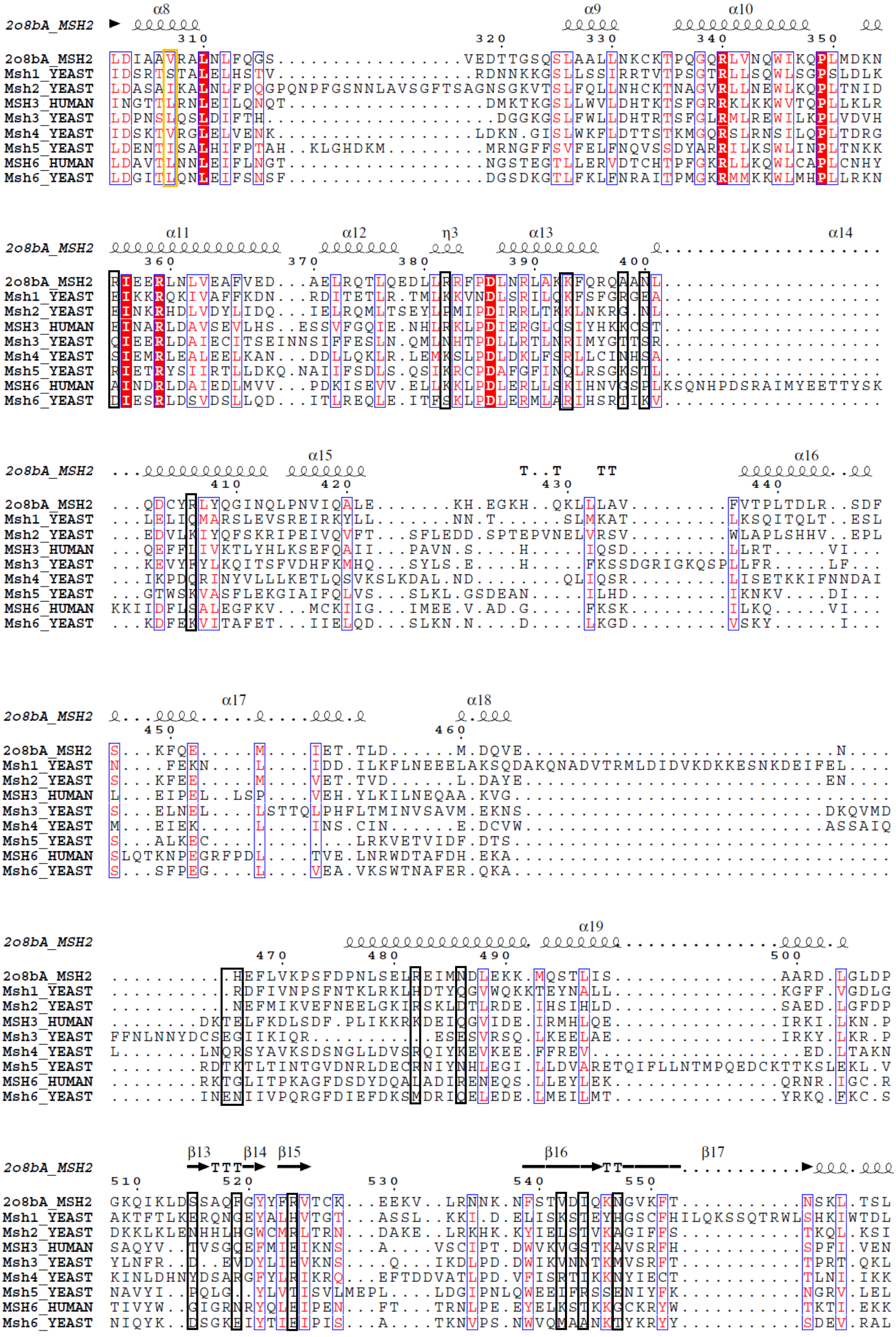

α8 α9 α10
2o8bA_MSH2
310 320 330 340 350
2o8bA_MSH2 LDIAAVRALNLFQGS.................VEDTTGSQSLAALLNKCKTPQGQRLVNQWIKQPLMDKN
Msh1_YEAST IDSRTSTALELHSTV.................RDNNKKGSLLSSIRRTVTPSGTRLLSQWLSGPSLDLK
Msh2_YEAST LDASAIKALNLFPQGPQNPFGSNNLAVSGFTSAGNSGKVTSLFQLLNHCKTNAGVRLLNEWLKQPLTNID
MSH3_HUMAN INGTTLRNLEILQNQT.................DMKTKGSLLWVLDHTKTSFGRRKLKKWVTQPLLKLR
Msh3_YEAST LDPNSLQSLDIFTH..................DGGKGSLFWLLDHTRTSFGLRMLREWILKPLVDVH
Msh4_YEAST IDSKTVRGLELVENK.................LDKN.GISLWKFLDTTSTKMGQRSLRNSILQPLTDRG
Msh5_YEAST LDENTISALHIFPTAH..KLGHDKM.........MRNGFFSVFELFNQVSSDYARRILKSWLINPLTNKK
MSH6_HUMAN LDAVTLNNLEIFLNGT.................NGSTEGTLLERVDTCHTPFGKRLLKQWLCAPLCNHY
Msh6_YEAST LDGITLQNLEIFSNSF.................DGSDKGTLFKLFNRAITPMGKRMMKKWLMHPLLRKN
α11 α12 η3 α13 α14
2o8bA_MSH2
360 370 380 390 400
2o8bA_MSH2 RIEERLNLVEAFVED...AELRQTLQEDLLRRFPDLNRLAKKFQRQAANL...................
Msh1_YEAST EIKKRQKIVAFFKDN...RDITETLR.TMLKKVNDLSRILQKFSFGRGEA...................
Msh2_YEAST EINKRHDLVDYLIDQ...IELRQMLTSEYLPMIPDIRRLTKKLNKRG.NL...................
MSH3_HUMAN EINARLDAVSEVLHS..ESSVFGQIE.NHLRKLPDIERGLCSIYHKKCST...................
Msh3_YEAST QIEERLDAIECITSEINNSIFFESLN.QMLNHTPDLLRTLNRIMYGTTSR...................
Msh4_YEAST SIEMRLEALEELKAN...DDLLQKLR.LEMKSLPDLDKLFSRLLCINHSA...................
Msh5_YEAST RIETRYSIIRTLLDKQ.NAIFSDLS.QSIKRCPDAFGFINQLRSGKSTL...................
MSH6_HUMAN AINDRLDAIEDLMVV...PDKISEVV.ELLKKLPDLERLLSKIHNVGSPLKSQNHPDSRAIMYEETTYSK
Msh6_YEAST DIESRLDSVDSLLQD...ITLREQLE.ITFSKLPDLERMLARIHSRTIKV...................
α15 α16
2o8bA_MSH2 T..T TT
410 420 430 440
2o8bA_MSH2 ...QDCYRLYQGINQLPNVIQALE.......KH.EGKH..QKLLLAV.........FVTPLTDLR..SDF
Msh1_YEAST ...LELIQMARSLEVSREIRKYLL.......NN.T.......SLMKAT.........LKSQITQLT..ESL
Msh2_YEAST ...EDVLKIYQFSKRIPEIVQVFT...SFLEDD.SPTEPVNELVRSV.........WLAPLSHHV..EPL
MSH3_HUMAN ...QEFFLIVKTLYHLKSEFQAII...PAVN.S....H.....IQSD.........LLRT.....VI...
Msh3_YEAST ...KEVYFYLKQITSFVDHFKMHQ...SYLS.E....H.....FKSSDGRIGKQSPLLFR.....LF...
Msh4_YEAST ...IKPDQRINYVLLLKETLQSVKSLKDAL.ND........QLIQSR.........LISETKKIFNNDAI
Msh5_YEAST ...GTWSKVASFLEKGIAIFQLVS...SLKL.GSDEAN.....ILHD.........IKNKV....DI...
MSH6_HUMAN KKIIDFLSALEGFKV...MCKIIG...IMEE.V.AD.G.....FKSK.........ILKQ.....VI...
Msh6_YEAST ...KDFEKVITAFET...IIELQD...SLKN.N....D.....LKGD.........VSKY......I...
α17 α18
2o8bA_MSH2
450 460
2o8bA_MSH2 S...KFQE.....M....IET.TLD......M.DQVE............................N....
Msh1_YEAST N....FEKN....L....IDD.ILKFLNEEELAKSQDAKQNADVTRMLDIDVKDKKESNKDEIFEL....
Msh2_YEAST S...KFEE.....M....VET.TVD......L.DAYE............................EN....
MSH3_HUMAN L...EIPEL..LSP....VEH.YLKILNEQAA.KVG..................................
Msh3_YEAST S...ELNEL....LSTTQLPHFLTMINVSAVM.EKNS............................DKQVMD
Msh4_YEAST M...EIEK.....L....INS.CIN......E.DCVW............................ASSAIQ
Msh5_YEAST S...ALKEC..............LRKVETVIDF.DTS..................................
MSH6_HUMAN SLQTKNPEGRFPDL....TVE.LNRWDTAFDH.EKA...................................
Msh6_YEAST S...SFPEG....L....VEA.VKSWTNAFER.QKA...................................
α19
2o8bA_MSH2
470 480 490 500
2o8bA_MSH2 ..........HEFLVKPSFDPNLSELREIMNDLEKK.MQSTLIS...............AARD.LGLDP
Msh1_YEAST ..........RDFIVNPSFNTKLRKLHDTYQGVWQKKTEYNALL...............KGFF.VGDLG
Msh2_YEAST ..........NEFMIKVEFNEELGKIRSKLDTLRDE.IHSIHLD...............SAED.LGFDP
MSH3_HUMAN ........DKTELFKDLSDF.PLIKKRKDEIQGVIDE.IRMHLQE...............IRKI.LKN.P
Msh3_YEAST FFNLNNYDCSEGIIKIQR..........ESESVRSQ.LKEELAE...............IRKY.LKR.P
Msh4_YEAST L.......LNQRSYAVKSDSNGLLDVSRQIYKEVKEE.FFREV...............ED.LTAKN
Msh5_YEAST ........RDTKTLTINTGVDNRLDECRNIYNHLEGI.LLDVARETQIFLLNTMPQEDCKTTKSLEKL.V
MSH6_HUMAN ........RKTGLITPKAGFDSDYDQALADIRENEQS.LLEYLEK...............QRNR.IGC.R
Msh6_YEAST ........INENIIVPQRGFDIEFDKSMDRIQELEDE.LMEILMT...............YRKQ.FKC.S
β13 β14 β15 β16 β17
2o8bA_MSH2 TTT TT
510 520 530 540 550
2o8bA_MSH2 GKQIKLDSSAQFGYYFRVTCK...EEKV..LRNNK.NFSTVDIQKNGVKFT...........NSKL.TSL
Msh1_YEAST AKTFTLKERQNGEYALHVTGT...ASSL..KKI.D.ELISKSTEYHGSCFHILQKSSQTRWLSHKIWTDL
Msh2_YEAST DKKLKLENHHLHGWCMRLTRN...DAKE..LRKHK.KYIELSTVKAGIFFS...........TKQL.KSI
MSH3_HUMAN SAQYV..TVSGQEFMIEIKNS...A.....VSCIPT.DWVKVGSTKAVSRFH...........SPFI.VEN
Msh3_YEAST YLNFR..D..EVDYLIEVKNS....Q....IKDLPD.DWIKVNNTKMVSRFT...........TPRT.QKL
Msh4_YEAST KINLDHNYDSARGFYLRIKRQ...EFTDDVATLPD.VFISRTIKKNYIECT...........TLNI.IKK
Msh5_YEAST NAVYI..PQLG..YLVTISVLMEPL...LDGIPNLQWEEIFRSSENIYFK...........NGRV.LEL
MSH6_HUMAN TIVYW..GIGRNRYQLEIPEN...FT...TRNLPE.EYELKSTKKGCKRYW...........TKTI.EKK
Msh6_YEAST NIQYK..DSGKEIYTIEIPIS...A....TKNVPS.NWVQMAANKTYKRYY...........SDEV.RAL

```
2o8bA_MSH2          α20                  η4                   α21                         β18
                 560            570            580            590            600            610            620

2o8bA_MSH2   NEEYTKNKTEYEEAQDA.IVKEIVNISSGYVEPMQ.TLNDVLAQLDAVVSFAHVSNGAPVPYVRPAI..L
Msh1_YEAST   GHELELLNLKIRNEEAN.IIDLFKRKFIDRSNEVR.QVATTLGYLDTLSSFAVLAN..ERNLVCPKV..D
Msh2_YEAST   ANETNILQKEYDKQQSA.LVREIINITLTYTPVFE.KLSLVLAHLDVIASFAHTSSYAPIPYIRPKLHPM
MSH3_HUMAN   YRHLNQLREQLVLDCSA.EWLDFLEKFSEHYHSLC.KAVHHLATVDCIFSLAKVAKQ..GDYCRPTVQ..
Msh3_YEAST   TQKLEYYKDLLIRESEL.QYKEFLNKITAEYTELR.KITLNLAQYDCILSLAATSCN..VNYVRPTFV..
Msh4_YEAST   NARLKEVMEEILLLSEE.TVDELLDKIATHISELF.MIAEAVAILDLVCSFTYNLK..ENNYTIPIF..T
Msh5_YEAST   DETYGDIYGA.ISDFEIEILFSLQEQILRRKTQLT.AYNILLSELEILLSFAQVSAE..RNYAEPQLV..
MSH6_HUMAN   LANLINAEERRDVSLKD.CMRRLFYNFDKNYKDWQ.SAVECIAVLDVLLCLANYSRGGDGPMCRPVIL..
Msh6_YEAST   ARSMAEAKEIHKTLEED.LKNRLCQKFDAHYNTIWMPTIQAISNIDCLLAITRTSEYLGAPSCRPTIV..
```

```
2o8bA_MSH2    TT                β19                          β20                  TT     β21      TT
                           630            640            650            660            670

2o8bA_MSH2   EKG........QGRIILKASRHACVEVQD....EIAFIPNDVYFE...........KDKQMFHIITGPNM
Msh1_YEAST   E..........SNKLEVVNGRHLMVEEGLSARSLETFTANNCELA.............KDNLWVITGPNM
Msh2_YEAST   DS.........ERRTHLISSRHPVLEMQD....DISFISNDVTLE...........SGKGDFLIITGPNM
MSH3_HUMAN   E..........ERKIVIKNGRHPVIDVLLG..EQDQYVPNNTDLS...........EDSERVMIITGPNM
Msh3_YEAST   NG.........QQAIIAKNARNPIIES.....LDVHYVPNDIMMS...........PENGKINIITGPNM
Msh4_YEAST   ............NNLLIRDSRHPLLEKV.....LKNFVPNTISST...........KHSSSLQIITGCNM
Msh5_YEAST   ED.........ECILEIINGRHALYET.....FLDNYIPNSTMIDGGLFSELSWCEQNKGRIIVVTGANA
MSH6_HUMAN   LPEDT......PPFLELKGSRHPCITKTF...FGDDFIPNDILIGCE....EEEQENGKAYCVLVTGPNM
Msh6_YEAST   DEVDSKTNTQLNGFLKFKSLRHPCFNLGAT..TAKDFIPNDIELG...........KEQPRLGLLTGANA
```

```
2o8bA_MSH2       α22                        β22       β23                   α23               TT
                  680            690            700            710            720            730            740

2o8bA_MSH2   GGKSTYIRQTGVIVLMAQIGCFVPCESAEVSIVDCILARVGAGDSQLKGVSTFMAEMLETASILRSATKD
Msh1_YEAST   GGKSTFLRQNAIIVILAQIGCFVPCSKARVGIVDKLFSRVGSADDLYNEMSTFMVEMIETSFILQGATER
Msh2_YEAST   GGKSTYIRQVGVISLMAQIGCFVPCEEAEIAIVDAILCRVGAGDSQLKGVSTFMVEILETASILKNASKN
MSH3_HUMAN   GGKSSYIKQVALITIMAQIGSYVPAEEATIGIVDGIFTRMGAADNIYKGRSTFMEELTDTAEIIRKATSQ
Msh3_YEAST   GGKSSYIRQVALLTIMAQIGSFVPAEEIRLSIFENVLTRIGAHDDIINGDSTFKVEMLDILHILKNCNKR
Msh4_YEAST   SGKSVYLKQVALICIMAQMGSGIPALYGSFPVFKRLHARVC.NDSMELTSSNFGFEMKEMAYFLDDINTE
Msh5_YEAST   SGKSVYLTQNGLIVYLAQIGCFVPAERARIGIADKILTRIRTQETVYKTQSSFLLDSQQMAKSLSLATEK
MSH6_HUMAN   GGKSTLMRQAGLLAVMAQMGCYVPAEVCRLTPIDRVFTRLGASDRIMSGESTFFVELSETASILMHATAH
Msh6_YEAST   AGKSTILRMACIAVIMAQMGCYVPCESAVLTPIDRIMTRLGANDNIMQGKSTFFVELAETKKILDMATNR
```

```
2o8bA_MSH2   β24                   α24                      β25       TT      α25
                      750            760            770            780            790            800

2o8bA_MSH2   SLIIIDELGRGTSTYDGFGLAWAISEYIATKIGAF.CMFATHFHEL.TALANQI...PT......VNNLH
Msh1_YEAST   SLAILDEIGRGTSGKEGISIAYATLKYLLENNQCR.TLFATHFGQELKQIIDNK...CSKGMSEKVKFYQ
Msh2_YEAST   SLIIVDELGRGTSTYDGFGLAWAIAEHIASKIGCF.ALFATHFHEL.TELSEKL...PN......VKNMH
MSH3_HUMAN   SLVILDELGRGTSTHDGIAIAYATLEYFIRDVKSL.TLFVTHYPPV.CELEKNYS..HQ......VGNYH
Msh3_YEAST   SLLLLDEVGRGTGTHDGIAISYALIKYFSELSDCPLILFTTHFPML.GEIK..S...PL......IRNYH
Msh4_YEAST   TLLILDELGRGSSIADGFCVSLAVTEHLLRTE.AT.VFLSTHFQDI.PKIMSKK...PA......VSHLH
Msh5_YEAST   SLILIDEYGKGTDILDGPSLFGSIMLNMSKSEKCPRIIACTHFHEL.FNENVLTENIKG......IKHYC
MSH6_HUMAN   SLVLVDELGRGTATFDGTAIANAVVKELAETIKCR.TLFSTHYHSL.VEDYSQN...VA......VRLGH
Msh6_YEAST   SLLVVDELGRGGSSSDGFAIAESVLHHVATHIQSL.GFFATHYGTL.ASSFKHH...PQ......VRPLK
```

```
2o8bA_MSH2               β26                         β27              α26            α27
                                             810            820            830            840            850

2o8bA_MSH2   VTA..............L....TTEETLTMLYQVK.KGVCDQSFGIHVAELANFPKHVIECAKQKALELE
Msh1_YEAST   SG...............ITD..LGGNNFCYNHKLK.PGICTKSDAIRVAELAGFPMEALKEARE......
Msh2_YEAST   VVAHIE.........KNLKEQKHDDEDITLLYKVE.PGISDQSFGIHVAEVVQFPEKIVKMAKRKANELD
MSH3_HUMAN   MGFLVSEDESKLD..PGA..AEQVPDFVTFLYQIT.RGIAARSYGLNVAKLADVPGEILKKAAHKSKELE
Msh3_YEAST   MDYVEE..........QK..TGEDWMSVIFLYKLK.KGLTYNSYGMNVAKLARLDKDIINRAFSISEELR
Msh4_YEAST   MDA.............VL...LNDNSVKMNYQLTQKSVAIENSGIRVVKKIF.NPDIIAEAYNIHSLLK
Msh5_YEAST   TDILISQKYNLLETAHVG..EDHESEGITFLFKVK.EGISKQSFGIYCAKVCGLSRDIVERAEELSRMIN
MSH6_HUMAN   MACMVE.........NEC..EDPSQETITFLYKFI.KGACPKSYGFNAARLANLPEEVIQKGHRKAREFE
Msh6_YEAST   MSILV..................DEATRNVTFLYKML.EGQSEGSFGMHVASMCGISKEIIDNAQIAADNLE
```

```
2o8bA_MSH2
                  860            870            880            890            900

2o8bA_MSH2   ..EFQYIGESQGYDIMEPAAKKCYLEREQGEKIIQ....EFLSK.......VKQMPFTEMSEENITIKLK
Msh1_YEAST   ......................................................................
Msh2_YEAST   ..DLKT....NN....EDLKKAKLSLQEVNEGNIRLKA.LLKEWIRKVKEEGLHDP.SKITEEASQHKIQ
MSH3_HUMAN   ..GLINT...........K.........RKRLK.................................YFA
Msh3_YEAST   ..KES...................INEDALK...................................LFS
Msh4_YEAST   IAKART....EN....EDS..............................NGVVDQ.KTINQMKRIHNLV
Msh5_YEAST   ..RGDDVVQ.QC....GNLTEKEMREFQKNQEIVK...............................KFL
MSH6_HUMAN   ..KMN......................QSLR...................................LFR
Msh6_YEAST   ..HTSRL...........VKERDLAANNLNGEVVSVPGGLQSDFVRIAYGDGLKNT.KLGSGE....GVL
```

*2o8bA_MSH2*

```
           910             920             930
             .               .               .
2o8bA_MSH2   QL.KA.....EVIAKNNS.FVN.....EIISRIK.VTT
Msh1_YEAST   ...............................ILG
Msh2_YEAST   ELLRAIA.NEPE.KENDN.YLK.....YIK...A.LLL
MSH3_HUMAN   KLWTMHN.AQDL.QKWTE.EFN.....MEE...T.QTS
Msh3_YEAST   SLKRILK.SDNI.TATDK.LAK.....LLS...L.DIH
Msh4_YEAST   AILKECAGNEKE.PLTLG.KLKEINSDFIE...N.FEE
Msh5_YEAST   SWD..LD.LETT.TTSEN.LRL.....KLK...N.FLR
MSH6_HUMAN   EVC..LA.SERS.TVDAEAVHK.....LLT...LIKEL
Msh6_YEAST   NYD..WN.IKR..NVLKS.LFS.....IID...D.LQS
```
