# Supplementary figures and images for: Structural Insights into Saccharomyces cerevisiae Msh4–Msh5 Complex Function Using Homology Modeling
Source: PLoS One. 2013 Nov 14;8(11):e78753. doi: 10.1371/journal.pone.0078753 (PMC3828297; doi:10.1371/journal.pone.0078753)

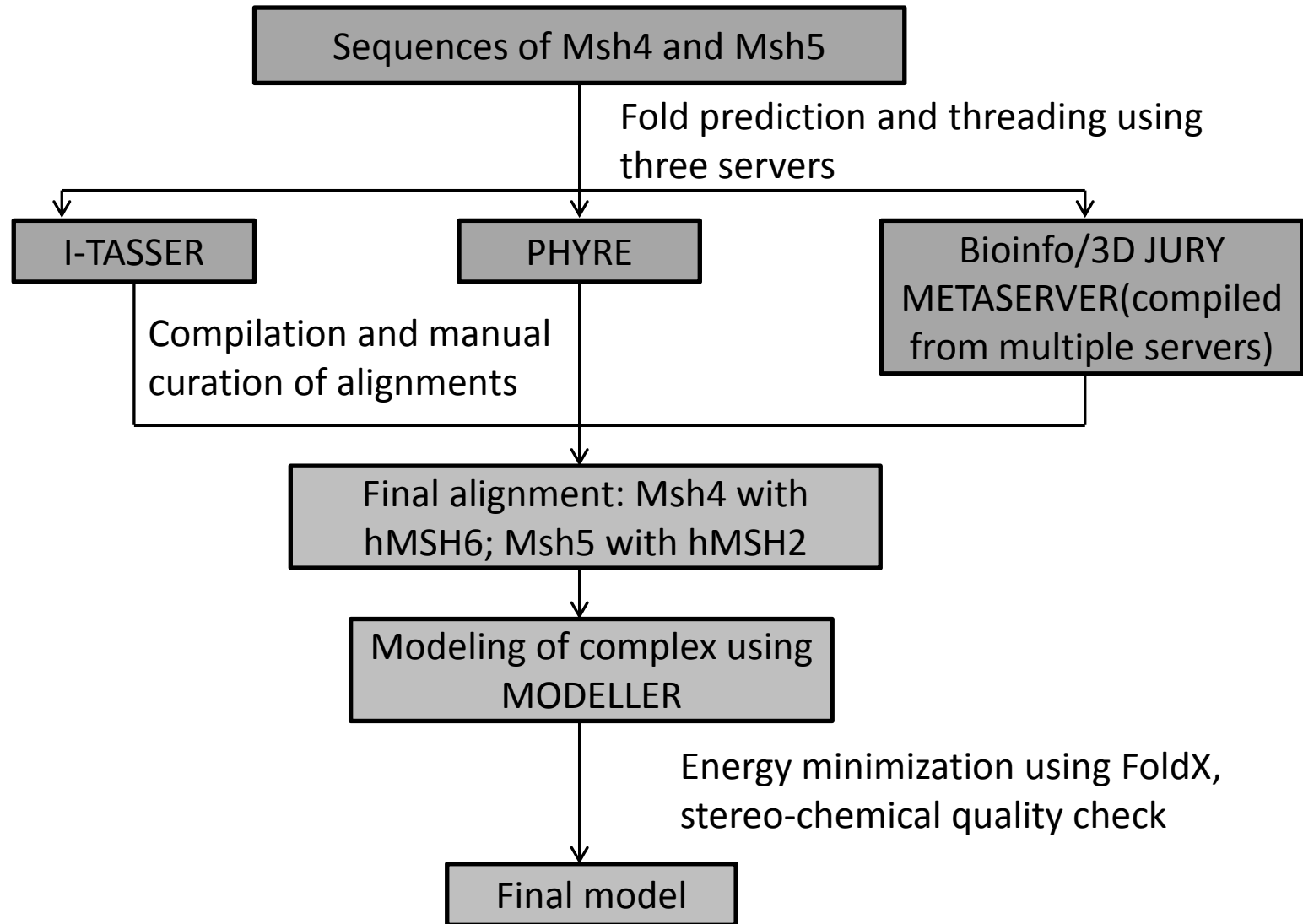

Supplement: Figure S4 — Protocol used to model Msh4–Msh5 structure. The sequences were submitted to three structure prediction servers and the alignment obtained from all these were compiled and manually curated to get final alignment which was provided to MODELER to build the structure. The structure was then energy minimized and checked for stereo-chemical quality (removal of short contacts). (PDF) [file pone.0078753.s004.pdf]
