# Supplementary material for: Structural Insights into Saccharomyces cerevisiae Msh4–Msh5 Complex Function Using Homology Modeling
Source: PLoS One. 2013 Nov 14;8(11):e78753. doi: 10.1371/journal.pone.0078753 (PMC3828297; doi:10.1371/journal.pone.0078753)
Supplement: Table S3 — Explanation for meiotic defects observed in msh4/5 mutants based on the homology model. (DOCX) [file pone.0078753.s007.docx]

| *msh4/5*  mutant | Residue number as per  model | SV (%) | Total Rf (cM) | Yeast two hybrid | Phenotype | Explanation |
| --- | --- | --- | --- | --- | --- | --- |
| Wild-type |  | 97 | 96.1 | + |  |  |
| *msh4* *D139A* | 909 | 31 | 38.6 | - | Null | Stabilization of helix four residues downstream is affected |
| *msh4* *G639A* | 1401 | 30 | 42.6 | - | Null | ø value corresponds to left handed helix which is not tolerated in case of Alanine mutation |
| *msh4* *Y143A* | 913 | 76.1 | 41.5 | n.d | Intermediate defect | Packing, cation pi interactions, side chain hydrogen bonding are affected |
| *msh4* *F194A* | 964 | 56.7 | 44.1 | n.d | Intermediate defect | Aromatic-aromatic interactions with Y206 and F251 affected |
| *msh4* *R456A* | 1223 | 61 | 40.5 | - | Intermediate defect | Stability of protein-DNA complex affected |
| *msh4* *L493A* | 1260 | 75 | 43.5 | - | Intermediate defect | No explanation could be provided |
| *msh4* *E276A* | 1044 | 88.9 | 53.2 | + | Crossover defect only | Packing of the residues affected due to smaller size of Alanine |
| *msh4* *F491A* | 1258 | 91 | 47.6 | - | Crossover defect only | Cation pi interaction with R468 affected |
| *msh4 N532A* | 1298 | 89.4 | 64.5 | n.d | Crossover defect only | Buried residue whose packing may affect DNA binding |
| *msh4* *R676W* | 1438 | 89.6 | 55.6 | + | Crossover defect only | Side chain interaction with E425, E428 lost |
|  |  |  |  |  |  |  |
| *msh5* *W298A* | 233 | 40.2 | 30.6 | - | Null | Aromatic interaction with F445, cation pi interaction with R312 is lost |
| *msh5* *D433A* | 368 | 47.3 | 37 | - | Null | Hydrogen bonding with side chain of D433 and main chain amide of N430 is lost |
| *msh5* *V488A* | 423 | 39.7 | 39.6 | - | Null | Packing affected which alters stability of protein-DNA complex |
| *msh5* *D527A* | 462 | 30.2 | 34.3 | - | Null | Stability of local structure is affected when the solvent exposed D527 is mutated to Alanine |
| *msh5* *G648A* | 583 | 33.3 | 34 | + | Null | Same as msh4 G639 |
| *msh5* *Y661A* | 596 | 45.8 | 33.6 | - | Null | Tyr residue is tightly packed which is affected since Ala is much smaller in size |
| *msh5* *R685W* | 620 | 36 | 35.2 | + | Null | Side chain interaction with D250, stability of β sheet affected |
| *msh5* *R436A* | 371 | 50.2 | 37.6 | - | Intermediate defect | Interaction with DNA affected |
| *msh5* *Y480A* | 415 | 67 | 37.8 | - | Intermediate defect | Aromatic-aromatic interactions with Y530, Y534 and Y486 and interaction with DNA affected |
| *msh5* *D532A* | 467 | 64.5 | 38.7 | - | Intermediate defect | no reason could be provided |
| *msh5* *L548A* | 483 | 50.2 | 36.1 | n.d | Intermediate defect | Alanine is a smaller residue and hence the local packing is affected |
| *msh5* *D680A* | 615 | 75 | 38.6 | - | Intermediate defect | Side chain interactions with K681, K716 lost |
| *msh5* *D76A* | 18 | 88 | 53.9 | - | Crossover defect only | No explanation could be provided |
| *msh5 D250A* | 189 | 91 | 60 | - | Crossover defect only | Side chain interaction with R685 affected |
| *msh5* *S416A* | 351 | 90.9 | 60 | n.d | Crossover defect only | DNA binding affected |
| *msh5 Y486A* | 421 | 93.8 | 62.9 | n.d | Crossover defect only | Aromatic-aromatic interactions lost |
| *msh5* *D539A* | 474 | 90.4 | 63.9 | + | Crossover defect only | No reason could be provided |

Spore viability (S.V), Recombination frequency (Rf) and yeast two hybrid data are from Nishant et al., 2010 [6]. More detailed explanations for the *msh4/5* mutants are provided in the text. n.d- Not determined.
